# Phytotoxic Terpenoids from *Ligularia cymbulifera* Roots

**DOI:** 10.3389/fpls.2016.02033

**Published:** 2017-01-09

**Authors:** Jia Chen, Guowei Zheng, Yu Zhang, Haji A. Aisa, Xiao-Jiang Hao

**Affiliations:** ^1^The Key Laboratory of Plant Resources and Chemistry of Arid Zone, Xinjiang Technical Institute of Physics and Chemistry, Chinese Academy of SciencesUrumqi, China; ^2^State Key Laboratory of Phytochemistry and Plant Resources in West China, Kunming Institute of Botany, Chinese Academy of SciencesKunming, China; ^3^Graduate School of Chinese Academy of SciencesBeijing, China; ^4^Germplasm Bank of Wild Species, Kunming Institute of Botany, Chinese Academy of SciencesKunming, China

**Keywords:** *Ligularia cymbulifera*, ligulacymirin A, ligulacymirin B, eremophilane sesquiterpenes, phytotoxic activity

## Abstract

*Ligularia cymbulifera* is one of the predominant species in the Hengduan Mountains, China, and has led to a decrease in the amount of forage grass in this area. However, little is known about the mechanism behind its predominance. In this study, two novel eremophilane sesquiterpenes, ligulacymirin A and B (**1** and **2**), together with seven other known terpenoids (**3–9**), were isolated from the roots of *L. cymbulifera*. The structures of **1** and **2** were determined by spectroscopic methods and single-crystal X-ray diffraction. Each compound showed phytotoxic activities against *Arabidopsis thaliana*, and each was detected and identified in rhizosphere soil by UHPLC-MS. Compound **3** was the most potent phytotoxin, showing remarkable inhibition against both seedling growth (EC_50_ = 30.33 ± 0.94 μg/mL) and seed germination (EC_50_ = 155.13 ± 0.52 μg/mL), with an average content in rhizosphere soil of 3.44 μg/g. These results indicate that terpenoids in *L. cymbulifera* roots might be released as phytotoxins in rhizosphere soil to interfere with neighboring plants.

## Introduction

Plants have developed complex eco-physiological strategies that allow them to outcompete neighboring plants. Releasing phytotoxins into the environment is thought to be one of the most important strategies influencing the dominance and succession of plants (Seigler, 1996). Phytotoxins are bioactive secondary metabolites that evolved in plants for defensive purposes, which exhibit strong phytotoxic effects on seed germination and the growth of other neighboring plant communities (Field et al., 2006). Many phytotoxic secondary metabolites are produced by plant roots, and their major mechanisms of release into the rhizosphere soil are root exudation and decomposition of plant root residue (Bertin et al., 2003). To shed light on these phytotoxins, it is important to detect and quantify them in rhizosphere soil (Macias et al., 2014). These phytotoxic secondary metabolites could offer interesting templates for potential agricultural applications, for example, as eco-friendly natural herbicides (Macías et al., 2008). Phytotoxins can be grouped into three main classes: terpenoids, N-containing compounds, and phenolic compounds (Huang et al., 2010). Eremophilane sesquiterpenes have been shown to be an important class of secondary metabolites responsible for phytotoxic activities (Andolfi et al., 2013; Masi et al., 2014; Miranda et al., 2015; Wang et al., 2015). Various skeletons of eremophilane sesquiterpenes have been identified as major secondary metabolites in the genus *Ligularia* (Yang et al., 2011; Kuroda et al., 2012; Saito, 2012; Tori, 2016), and some have been reported to display phytotoxicity (Cantrell et al., 2007).

**Figure 1:**
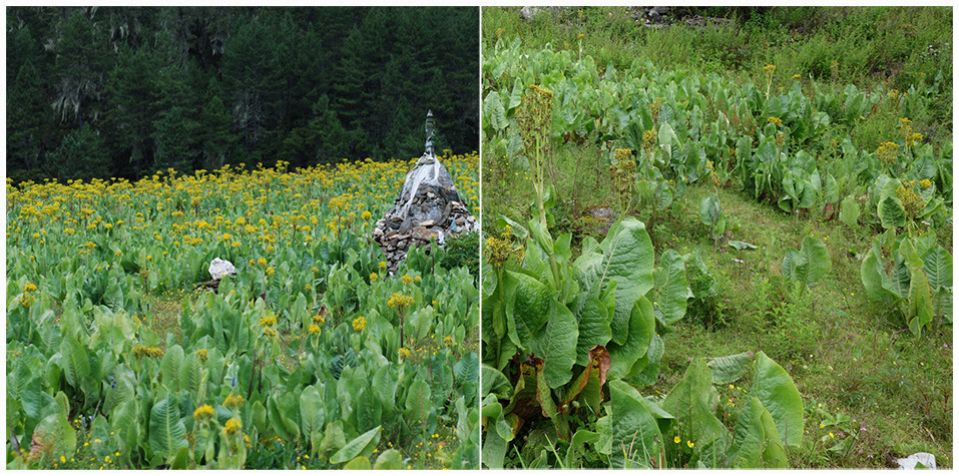
**The habitat of *L. cymbulifera* in moist grassland in Zhongdian, Yunnan**.

*Ligularia cymbulifera* (W. W. Smith) Hand. Mazz, belonging to the Asteraceae family, is one of the predominant species in the Hengduan Mountains, China. It is a perennial herb that grows at high density in moist grassland at altitudes from 3000 to 4800 m, being especially abundant in Zhongdian, Yunnan (Hanai et al., 2005). The population of this plant has recently exhibited a continuous increase in grassland, causing a decrease in the amount of forage grass in this area (Figure 1). It was reported that eremophilane sesquiterpenes, bisabolane sesquiterpenes, and pyrrolizidine alkaloids are the main secondary metabolites of this plant (Hanai et al., 2005; Liu et al., 2006, 2008; Wu et al., 2012). In addition, furanoeremophilan-10β-ol (**3**) was found to be an abundant eremophilane sesquiterpene produced by *L. cymbulifera*, and was thought to be a defensive agent that helps this species to gain an ecological advantage (Kuroda et al., 2012). However, no evidence in support of the above hypotheses has been presented thus far (Iida et al., 2007). Accordingly, we investigated whether secondary metabolites in *L. cymbulifera* could play phytotoxic roles and, if so, what mechanisms of action are involved.

In this study, we isolated nine terpenoids, including two novel eremophilane sesquiterpene derivatives, and deduced their structures. We also evaluated their phytotoxic potential, and further detected and identified all these phytotoxic compounds in rhizosphere soil. The results indicated that phytotoxic terpenoids in *L. cymbulifera* might be released into rhizosphere soil, and might provide this species with a competitive advantage by interfering with the germination and root elongation of neighboring plants. To the best of our knowledge, this is the first report on phytotoxic terpenoids in the roots of *L. cymbulifera*, which may provide new insights into the successful competitive mechanisms of this plant.

## Materials and methods

### Plant material

The roots of *L. cymbulifera* were collected in Zhongdian, Yunnan Province, China, in September 2015, and identified and photographed by Associate Professor Yang Liu of Kunming Institute of Botany (KIB), Chinese Academy of Sciences (CAS). A voucher specimen (KIB H20150913) has been deposited in the State Key Laboratory of Phytochemistry and Plant Resources in West China, KIB, CAS. Seeds of *A. thaliana* were of the Columbia wild ecotype.

### General experimental procedures

Optical rotation (OR) values were measured with a Jasco P-1020 (Jasco International, Tokyo, Japan) automatic digital spectropolarimeter. Ultraviolet (UV) spectral data were obtained using a Shimadzu UV-2401PC spectrophotometer (Shimadzu, Tokyo, Japan). Infrared spectroscopy (IR) was performed using a Bruker Tensor 27 FT-IR spectrometer (Bruker Optics, Ettlingen, Germany) with KBr pellets. One-dimensional (1D) and two-dimensional (2D) nuclear magnetic resonance (NMR) spectra were obtained in CD_3_OD or DMSO-*d*_6_ on a Bruker AVANCE III 500 MHz spectrometer (Bruker, Karlsruhe, Germany) with tetramethylsilane as an internal standard. Chemical shifts (δ) are expressed in ppm with reference to the solvent signals. Electrospray ionization mass spectrometry (ESI-MS) and high-resolution (HR)-ESI-MS were carried out on a Waters Xevo TQ-S mass spectrometer (Waters Corp., Milford, MA, USA). X-ray diffraction data collection was performed on a Bruker SMART APEX CCD (Bruker, Karlsruhe, Germany) crystallography system. Normal-pressure column chromatography (CC) was performed on either silica gel (100–200 mesh and 300–400 mesh; Qingdao Marine Chemical Inc., Qingdao, China) or Sephadex LH-20 (40–70 μm; GE Healthcare Bio-Sciences AB, Uppsala, Sweden). Preparative medium-pressure liquid chromatography (MPLC) was performed on a Buchi Separate system using MCI-gel CHP 20P (70–150 μm; Mitsubishi Chemical Industries Ltd., Tokyo, Japan). Preparative high-performance liquid chromatography (HPLC) was performed on an Agilent 1200 liquid chromatography system (Agilent Technologies, Santa Clara, CA, USA) equipped with an XSELECT CSH Prep C_18_ column (5 μm, 19 × 150 mm i.d.; Waters, Wexford, Ireland) and diode array detector (DAD). Fractions were monitored and analyzed by thin-layer chromatography (TLC) (GF_254_; Qingdao Marine Chemical Inc., Qingdao, China); spots were visualized under UV_254_ illumination and/or by heating silica gel plates dipped into 5% H_2_SO_4_ in ethanol. All solvents used for extraction and isolation were distilled at their boiling point range prior to use. HPLC-grade acetonitrile and formic acid were from Fisher Scientific (Loughborough, UK). Ultrapure water was prepared by a Milli-Q water purification system (Millipore, Bedford, MA, USA).

### Extraction and isolation of terpenoids

Air-dried roots of *L. cymbulifera* (15.0 kg) were powdered and extracted with 75% MeOH (v/v, 3 × 15 L) at 75°C under reflux three times (6 h each). The MeOH extracts were filtered and the solvent was evaporated under a vacuum to afford a crude MeOH extract (1300 g). The crude extract was then suspended in partition between H_2_O (4 L) and ethyl acetate (EtOAc) (3 × 4 L), and the EtOAc fraction (600 g) was subjected to CC over silica gel (100–200 mesh, 1600 g) eluting with dichloromethane (CH_2_Cl_2_)/acetone (Me_2_CO) (1:0; 9:1; 1:1; 0:1, v/v) to afford five fractions, A–E. Fraction A (CH_2_Cl_2_:Me_2_CO = 1:0, 180 g) was further separated by CC on a silica gel (300–400 mesh) using petroleum ether (PE) and Me_2_CO (100:1–20:1, v/v) to yield five fractions (Fr. A1–A5). Fr. A2 (5.6 g) was subjected to MPLC (MCI gel) using MeOH/H_2_O (20:80–80:20) and finally purified by Sephadex LH-20 (MeOH) and HPLC to give compounds **4** (167 mg), **5** (158 mg), **6** (210 mg), **7** (480 mg), and **9** (950 mg). Fraction B (CH_2_Cl_2_:Me_2_CO = 9:1, 127 g) was also subjected to MPLC (MCI gel) using MeOH/H_2_O (40:60–80:20) to yield five main fractions (Fr. B1–B5). Fr. B1 (MeOH:H_2_O = 40:60, 3 g) was purified by Sephadex LH-20 chromatography (MeOH) and then recrystallized to yield compound **8** (220 mg) and compound **3** (960 mg). Fr. B2 (MeOH:H_2_O = 60:40, 6 g) was separated by CC on a silica gel (300–400 mesh) using PE/Me_2_CO (20:1), and then further purified by preparative HPLC (XSELECT CSH Prep C_18_ column, 5 μm, 19 × 150 mm i.d.; Waters) using 30% aqueous acetonitrile (v/v) at a flow rate of 10 mL/min to afford compound **1** (retention time (*t*_R_) of 23 min, 680 mg) and compound **2** (*t*_R_ of 18 min, 920 mg).

### Data of the two novel eremophilane sesquiterpenes

*Ligulacymirin A (**1**)*. Colorless cubic crystals (MeOH); Mp: 175–176°C; ${\left[\alpha \right]}_{\text{D}}^{20}$ +20 (*c* 0.17, MeOH) (Figure S9); UV (MeOH) λ_max_ (log ε): 203.6 (3.62) nm (Figure S7); IR (KBr) ν_max_: 5323, 3386, 2964, 2934, 2913, 2866, 1778, 1445, 1380, 1325, 1245, 1223, 1131, 1112, 1077, 1040, 1023, 1012, 913, 863 cm^−1^ (Figure S8); for ^1^H and ^13^C NMR (500 MHz, DMSO-*d*_6_) spectroscopic data, see Table 1 (Figures S1–S6); positive ESIMS: *m/z* 343 [M+Na]^+^; positive HRESIMS *m*/*z* 359.1627 (calcd for C_19_H_28_O_4_K^+^, 359.1619) (Figure S10).

**Table 1:** **^1^H and ^13^C NMR (500 MHz) spectroscopic data of compounds1 and 2 (in DMSO-*d*_6_) and compound 3 (in CD_3_OD) (δ in ppm, *J* in Hz)^a^**.

**No**.	**1**		**2**		**3**	
	**δ_C_**	**δ_H_**	**δ_C_**	**δ_H_**	**δ_C_**	**δ_H_**
1α	33.7	1.23 (m)	34.1	1.27 (m)	35.1	1.46 (m)
1β		1.54 (m)		1.45 (d, *J* = 12.0, 5.6)		1.82 (td, *J* = 12.9, 5.0)
2α	21.9	1.42 (s)	21.9	1.37 (s)	23.5	1.59 (m)
2β		1.52 (m)		1.51 (d, *J* = 19.1, 6.7)		1.72 (m)
3α	29.0	1.33 (m)	29.0	1.29 (d, *J* = 12.7)	30.4	1.41 (m)
3β		1.21 (m)		1.25 (m)		1.38 (m)
4	32.5	1.35 (m)	32.6	1.49 (m)	34.3	1.45 (m)
5	39.5		38.9		42.7	
6α	31.6	2.04 (d, *J* = 17.4)	31.5	1.95 (d, *J* = 16.9)	28.6	2.42 (d, *J* = 16.3)
6β		1.85 (d, *J* = 17.4)		1.84 (d, *J* = 16.9)		2.26 (d, *J* = 16.3)
7	129.6		130.3		116.2	
8	128.3		125.8		148.7	
9α	38.5	2.41 (d, *J* = 18.4)	35.7	2.05 (d, *J* = 18.4)	34.0	3.14 (d, *J* = 17.4)
9β		1.58 (d, *J* = 18.4)		1.66 (d, *J* = 18.4)		2.36 (d, *J* = 17.4)
10	70.6		70.8		75.6	
11	85.0		82.7		120.5	
12	78.6	3.77 (d, *J* = 4.9)	78.6	3.69 (d, *J* = 5.2)	138.5	7.07 (s)
13	14.6	1.30 (s)	19.1	1.36 (s)	8.1	1.91 (d, *J* = 1.3)
14	14.6	0.79 (s)	14.8	0.78 (s)	15.4	0.99 (s)
15	16.0	0.73 (d, *J* = 6.5)	16.2	0.70 (d, *J* = 6.5)	16.5	0.81(d, *J* = 6.2)
16	40.6	2.08 (s)	39.0	2.38 (s)		
17	48.1		44.9			
18	180.3		178.7			
19	16.4	1.10 (s)	18.4	1.04 (s)		
OH-10		4.04 (s)		4.07 (s)		
OH-12		5.70 (d, *J* = 5.0)		5.59 (d, *J* = 5.2)		

a *The assignments were based on distortionless enhancement by polarization transfer (DEPT) and 2D NMR experiments*.

*Ligulacymirin B (**2**)*. Colorless crystals (MeOH); Mp: 180–182°C; ${\left[\alpha \right]}_{\text{D}}^{20}$ +24.3 (*c* 0.1, MeOH) (Figure S19); UV (MeOH) λ_max_ (log ε): 204.5 (3.62) (Figure S17); IR (KBr) ν_max_: 3489, 3422, 2967, 2928, 2908, 2879, 1746, 1630, 1449, 1381, 1333, 1306, 1278, 1228, 1189, 1099, 1048, 1032, 1011, 917 cm^−1^ (Figure S18); for ^1^H and ^13^C NMR (500 MHz, DMSO-*d*_6_) spectroscopic data, see Table 1 (Figures S11–S16); positive ESIMS: *m/z* 343 [M+Na]^+^; positive HRESIMS *m*/*z* 359.1623 (calcd for C_19_H_28_O_4_ K^+^, 359.1619) (Figure S20).

### Absolute structures of two novel eremophilane sesquiterpenes analysis by single-crystal X-ray diffraction

Colorless crystals of **1** and **2** were obtained in MeOH at room temperature. Crystallographic data were collected at 100 K on a Bruker APEX DUO diffractometer with APEX II CCD, using CuKα radiation. All calculations were performed using the SHELXS-97 program and refined by full-matrix least-squares refinements based on F^2^ with SHELXL-97. The absolute configurations of **1** and **2** were analyzed using Hooft methods. Crystallographic data for the reported structures have been deposited with the Cambridge Crystallographic Data Center as supplementary publication deposition number CCDC 1475236 for compound **1** and CCDC 1475237 for compound **2**. Copies of these data can be obtained free of charge from the Cambridge Crystallographic Data Center via http://www.ccdc.cam.ac.uk/data_request/cif.

Crystal data for **1**: C_19_H_28_O_4_, *M* = 320.41, orthorhombic, size 0.95 × 0.70 × 0.50 mm^3^, *a* = 7.9425 (2) Å, *b* = 10.8992 (2) Å, *c* = 19.3930 (4) Å, α = 90.00°, β = 90.00°, γ = 90.00°, *V* = 1678.79 (6) Å^3^, *T* = 100(2) K, space group *P*212121, *Z* = 4, μ (CuKα) = 0.701 mm^−1^, 9417 reflections measured, 2878 independent reflections (*R*_*int*_ = 0.0301). The final *R*_1_ values were 0.0340 (I > 2σ (*I*)). The final *wR* (*F*^2^) values were 0.0849 (*I* > 2σ (*I*)). The final *R*_1_ values were 0.0340 (all data). The final *wR* (*F*^2^) values were 0.0850 (all data). The goodness of fit on *F*^2^ was 1.139. Flack parameter = 0.12 (16). The Hooft parameter is 0.11(4) for 1168 Bijvoet pairs.

Crystal data for **2**: C_19_H_28_O_4_, *M* = 320.41, Monoclinic, size 0.970 × 0.380 × 0.260 mm^3^, *a* = 7.8489 (6) Å, *b* = 10.8350(8) Å, *c* = 10.2850 (8) Å, α = 90°, β = 104.762 (2)°, γ = 90°, *V* = 845.79(11) Å3, *T* = 100(2) K, space group P21, *Z* = 2, μ (CuKα) = 0.696 mm^−1^, 8987 reflections measured, 2924 independent reflections (*R*_*int*_ = 0.0362). The final *R*_1_ values were 0.0300 (*I* > 2σ (*I*)). The final *wR* (*F*^2^) values were 0.0807 (*I* > 2σ (*I*)). The final *R*_1_ values were 0.0300 (all data). The final *wR* (*F*^2^) values were 0.0807 (all data). The goodness of fit on *F*^2^ was 1.089. Flack parameter = 0.06 (5).

### Seed sterilization and plant growth

*A. thaliana* seeds were surface-sterilized with ethanol (75%, v/v) for 2 min and sodium hypochlorite (5%, v/v) for 2 min, and then rinsed three times with sterile distilled water. The surface-sterilized seeds were cold-stratified for 3 days at 4°C before use. The seeds were then sown on MS agar plates that contained 0.4% gellan gum (G1910; Sigma-Aldrich) and 1% sucrose. These glass Petri dishes (9 cm) were placed vertically for growth, and the conditions of the growth chamber were 23/18°C, a 12/12-h light/dark cycle, photosynthetic photon flux density of 150 μM·m^−2^·s^−1^, and relative humidity of 65%.

### Seed germination bioassay

The phytotoxic effects of the nine compounds were evaluated as described by Zheng et al., with minor modifications (Zheng et al., 2012). The initial solvent carriers (100 mg/mL) of compounds **1–5** and **7**–**9** were prepared using methanol, and that of compound **6** was prepared using dimethylsulfoxide (DMSO). After the sterilized MS medium had been cooled to 50°C, tested compounds were added to the medium to obtain the final serial concentrations. To assess the toxic effects of methanol or DMSO, MS medium containing 0.8% MeOH or DMSO (v/v) was used as a control. Three replicates were set for each treatment; in each replicate, 20 surface-sterilized seeds were sown equidistantly on MS medium. Subsequently, Petri dishes were sealed with Parafilm to retard moisture loss and then placed in the growth chamber. The germination rate of seeds was determined after 7 days, when over 95% of the control seeds had germinated, using emergence of the radicle (≥ 1 mm) as the index of germination. The seed germination inhibition (*I*_G_) was evaluated using the following equation: *I*_G_% = (1−*N*_T_/*N*) × 100. *N*_T_ is the number of germinated seeds for each treatment and *N* is the number of seeds used in the bioassay.

### Root elongation and determination of root death

To test the effects of compounds **1**–**9** on *A. thaliana* root elongation, seeds of this species were pretreated as described above. Compounds **1**–**9** were each assayed at different concentrations. Three replicates, with 20 seeds each, were set for each treatment, and 7 days after germination, the root length of each seedling was measured and recorded using electronic calipers. The percentage of growth inhibition of root length (*I*_R_) was calculated using the following equation: *I*_R_% = (1−*T*/*C*) × 100. *T* is the average root length (cm) of treated seeds and *C* is the average root length (cm) of the control. Seeds that produced a radicle but no coleoptile were scored as zero. To detect root death, roots of *A. thaliana* were stained with 5 μg/mL fluorescein diacetate (FDA; Sigma-Aldrich) for 5 min, and then rinsed three times with MS liquid medium. After staining and rinsing, the roots were observed under a confocal laser scanning microscope (FV-1000; Olympus, Tokyo, Japan). FDA fluorescence decreased as the dye leaked from dead cells.

### Rhizosphere soil sample

The rhizosphere soil of *L. cymbulifera* was collected in Zhongdian, Yunnan Province, China, in August 2016. The plants (ca. 60–80 cm in height) were randomly collected and carefully uprooted, and the rhizosphere soil was shaken off the roots. The soil was picked and crushed, and residues were then removed with a sieve (30 mesh). Three replicated sieved soil samples (100 g each) were stored at 75% MeOH (300 mL) for 3 days at room temperature, and then extracted ultrasonically at 75°C for 60 min. The extracts were filtered and concentrated in a vacuum and was then dissolved in MeOH (10 mL). The solution was centrifuged at 12,000 rpm for 10 min, the supernatant was passed through a 0.45 μm nylon membrane filter and then analyzed by ultra-high-performance liquid chromatography-mass spectrometry (UHPLC-MS).

### UHPLC-MS equipment and conditions

The LC analysis was carried out using an Agilent 1290 Infinity Series UHPLC system comprising a quaternary pump (G4204A, USA), an autosampler (G4226A, USA), a column compartment (G1316C, USA), and a DAD. Samples were separated on a Phenomenex Kinetex C18 column (1.7 μm, 2.1 × 100 mm i.d.; Phenomenex, Torrance, CA, USA) at room temperature. The mobile phase consisted of water containing 0.1% formic acid (A) and acetonitrile (B) and the elution gradient was set as follows: 32% B (0 min), 38% B (12 min), 85% B (15 min), 95% B (18 min). The mobile phase flow rate was 350 μL/min and the injected volume was set at 2 μL of standard and 5 μL of MeOH extracts of rhizosphere soil.

For the LC-ESI-MS^n^ experiments, a quadrupole time-of-flight high-resolution mass spectrometer (Q-TOF LC/MS 6540 series; Agilent Technologies) was connected to the UHPLC instrument via an ESI interface. The data were acquired using Mass Hunter workstation software. Detection was performed in positive ESI mode and the full scan mass range was set from m/z 100 to m/z 700. The MS parameters were optimized as follows: the fragment voltage was set at 135 V; the capillary was set at 3500 V; the skimmer was set at 65 V; and nitrogen was used as the drying (350°C, 6 L/min) and nebulizing (25 psi) gas.

### Identification of potential phytotoxins and quantification of compound 3 in rhizosphere soil by UHPLC-MS

Identification of compounds **1**–**9** in the rhizosphere soil samples of *L. cymbulifera* was undertaken by UHPLC-MS. Identification of potential phytotoxins was performed by comparing the retention times and MS/MS data with those of standards. Quantification of **3** in the rhizosphere soil was also undertaken using the same UHPLC method, with the isolated authentic sample as an external standard. Samples were also prepared in the same way as rhizosphere soil sample described above. For quantification, a calibration curve for **3** was prepared. Triplicate injections were carried out at four concentrations (1, 5, 20, 50 μg/mL), and standard curves were constructed by the linear regression method. The equation and correlation coefficient obtained from the linearity study for **3** were as follows: *y* = 2.492*x*+21.1673 (*r*^2^ = 0.9997).

### Statistical analysis

Each treatment was conducted with three replicates in a completely randomized design. The data on the inhibition of seed germination and root elongation are expressed as mean ± standard deviation (SD). The values of effective concentration producing 50% inhibition (EC_50_) were calculated using SPSS.

## Results and discussion

### Structural elucidation of secondary metabolites

**Figure 2:**
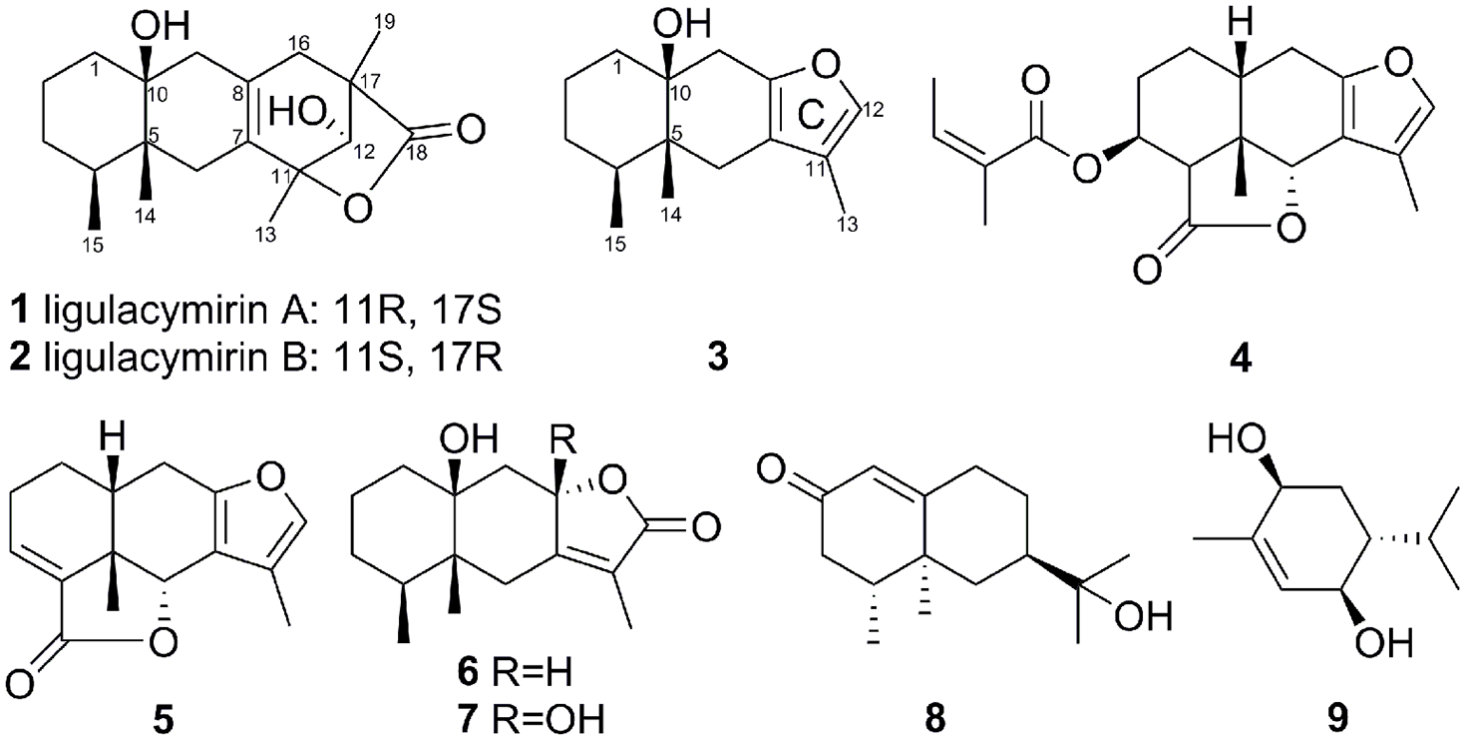
**Structures of compounds 1–9 isolated from the roots of *L. cymbulifera***.

Compounds **1** (680 mg), **2** (920 mg), **3** (960 mg), **4** (167 mg), **5** (158 mg), **6** (210 mg), **7** (480 mg) **8** (220 g), and **9** (950 mg) were isolated from the MeOH extracts of the air-dried roots of *L. cymbulifera* (Figure 2).

*Ligulacymirin A* (**1**) was obtained as optically active colorless cubic crystals from MeOH. Its molecular formula C_19_H_28_O_4_ was determined on the basis of the positive HRESIMS at *m/z*359.1627 (calcd for C_19_H_28_O_4_K^+^, 359.1619), corresponding to six degrees of unsaturation. The IR spectrum indicated the presence of hydroxyl (3386 cm^−1^) and lactone (1778 cm^−1^) groups. The ^13^C NMR (Table 1) and DEPT spectra of **1** exhibited signals for 19 carbons. Among these 19 carbons, a tetra-substituted double bond at δ_*C*_ 128.3 (C-8), 129.6 (C-7), and a carboxyl at δ_*C*_ 180.3 (C-18) were occupied with two degrees of unsaturation. Thus, the remaining four degrees of unsaturation indicated that **1** is a compound with four rings. Upon careful comparison of the NMR data of **1** with furanoeremophilan-10β-ol (**3**) (Table 1), a typical eremophilane sesquiterpene isolated from the same plant, the three methyl group signals at δ_H_ 0.73 (d, *J* = 6.5 Hz, H_3_-15), 0.79 (s, H_3_-14), and 1.30 (s, H_3_-13), which are characteristic of eremophilane sesquiterpenes, were observed. These data suggested that compound **1** is an eremophilane sesquiterpene derivative with the skeleton with 19 carbons.

**Figure 3:**
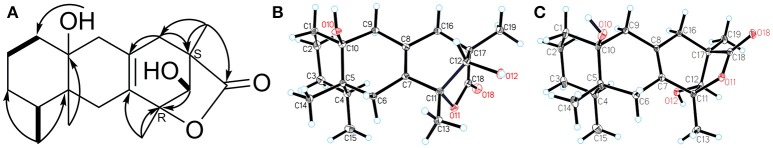
**A** ^1^H-^1^H COSY (bold) and key HMBC correlations (from H to C) of ligulacymirin **A** (**1**); **B** X-ray crystallographic structure of compound ligulacymirin **A** (**1**); **C** X-ray crystallographic structure of compound ligulacymirin **B** (**2**).

In the ^1^H-^1^H COSY spectrum, the cross peaks between δ_H_ 5.70 (d, *J* = 5.0, OH-12) and δ_H_ 3.77(d, *J* = 4.9, H_1_-12) suggested one free hydroxyl group link to C-12 (Figure 3). The HMBC correlations H-16/C-8, C-17; Me-19/C-16, C-17, C-18; Me-13/C-7, C-11; and H-12/C-11 revealed a ring C connected from C-7 to C-11 and C-8 to C-16, while δ_H_1.10 (s, H_3_-19) and 1.30 (s, H_3_-13) were located at C-19 and C-13, respectively (Figure 3). By analysis of NMR spectra of compounds **1** and **3**, the major difference between them was that the furan ring commonly appearing in eremophilane sesquiterpenes was clearly absent in **1** (Figure 2). Another free hydroxyl group at δ_H_ 4.04 (s, 10-OH) was located at C-10 due to the significant HMBC correlations from OH-10 to C-1. In addition, carboxyl at δ_*C*_ 180.3 (C-18) indicated a lactone moiety, which linked to C-11 through an ester bridge to occupy the last degrees of unsaturation. Thus, the planar structure of **1** was thus identified as shown in Figure 2. This assignment is in full agreement with the result of the X-ray crystallography (Figure 3). The absolute configuration of compound **1** was definitively determined to be 4S, 5R, 10S, 11R, 12S, 17S, and it was named ligulacymirin A. It is noteworthy that **1** is a novel eremophilane derivative with the skeleton with 19 carbons featuring an unusual 6/6/6/5 ring system.

By HRESIMS analysis, ligulacymirin B (**2**) showed the same molecular formula, C_19_H_28_O_4_, as ligulacymirin A. The 1D and 2D NMR spectra of **2** closely resemble those of **1**, clearly revealing that the planar structure of **2** was the same as that of **1** (Table 1). A single crystal of **2** was also obtained from MeOH and analyzed by X-ray crystallography (Figure 3) to confirm unambiguously the absolute configuration of **2**, which was assigned to be 4S, 5R, 10S, 11S, 12S, 17R, named ligulacymirin B. Ligulacymirin A and B are thus a pair of isomers.

The known compounds **3**–**9** were identified as furanoeremophilan-10β-ol (**3**) (Jennings et al., 1976); 3β-angeloyloxyeremophila-7,11-dien-14β,6α-olide (**4**) (Li et al., 2004); furanoeremophil-3-*en*-14,6α-olide (**5**) (Kuroda et al., 1982); 10β-dihydroxyeremophilenolide (**6**) (Aclinqu et al., 1991); 8β,10β-dihydroxyeremophilenolide (**7**) (Kojima et al., 1997); 11-hydroxyvalenc-1(10)-*en*-2-one (**8**) (Savona et al., 1987); and (*3R*,*4R*,*6S*)-3,6-dihydroxy-1-menthene (**9**) (Cuenca et al., 1991), respectively, on the basis that their HRESI-MS and NMR data were consistent with the literature.

### Terpenoids isolated from *L. cymbulifera* showed phytotoxic activity on *A. thaliana*

**Figure 4:**
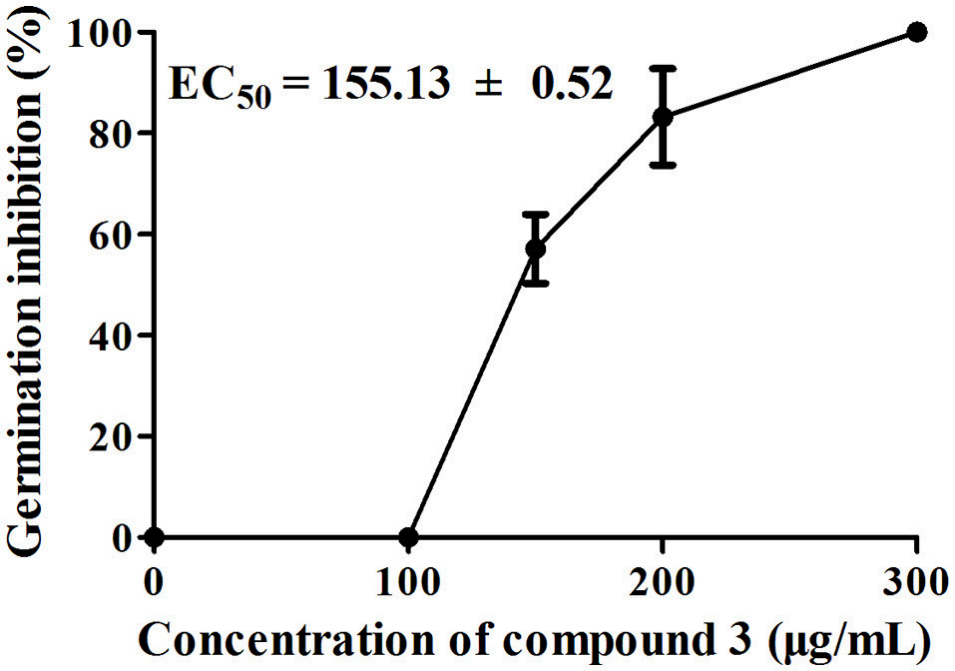
**Effects of compound 3 on *A. thaliana* seed germination after 7 days of treatment**. There were three replicates for each plate; error bars represent standard error of the mean, *n* = 3.

Among the nine compounds tested for the inhibition of *A. thaliana* seed germination, compound **3** showed the highest inhibitory activity, with an EC_50_ value of 155.13 ± 0.52 μg/mL, in a concentration-dependent manner from 100 to 300 μg/mL (Figure 4). Moreover, the rates of inhibition of seed germination for compound **7** were 11.75 and 93.64% at concentrations of 400 and 600 μg/mL, respectively; the rates of inhibition of seed germination by compound **9** were 59.11 and 93.06% at concentrations of 400 and 800 μg/mL, respectively. Compound **8** did not show any effect on seed germination at a concentration of 400 μg/mL, and the inhibition was 97.21% at a concentration of 800 μg/mL. Compounds **1**, **2**, and **4**–**6** displayed no inhibitory activity even at a concentration of 800 μg/mL.

**Figure 5:**
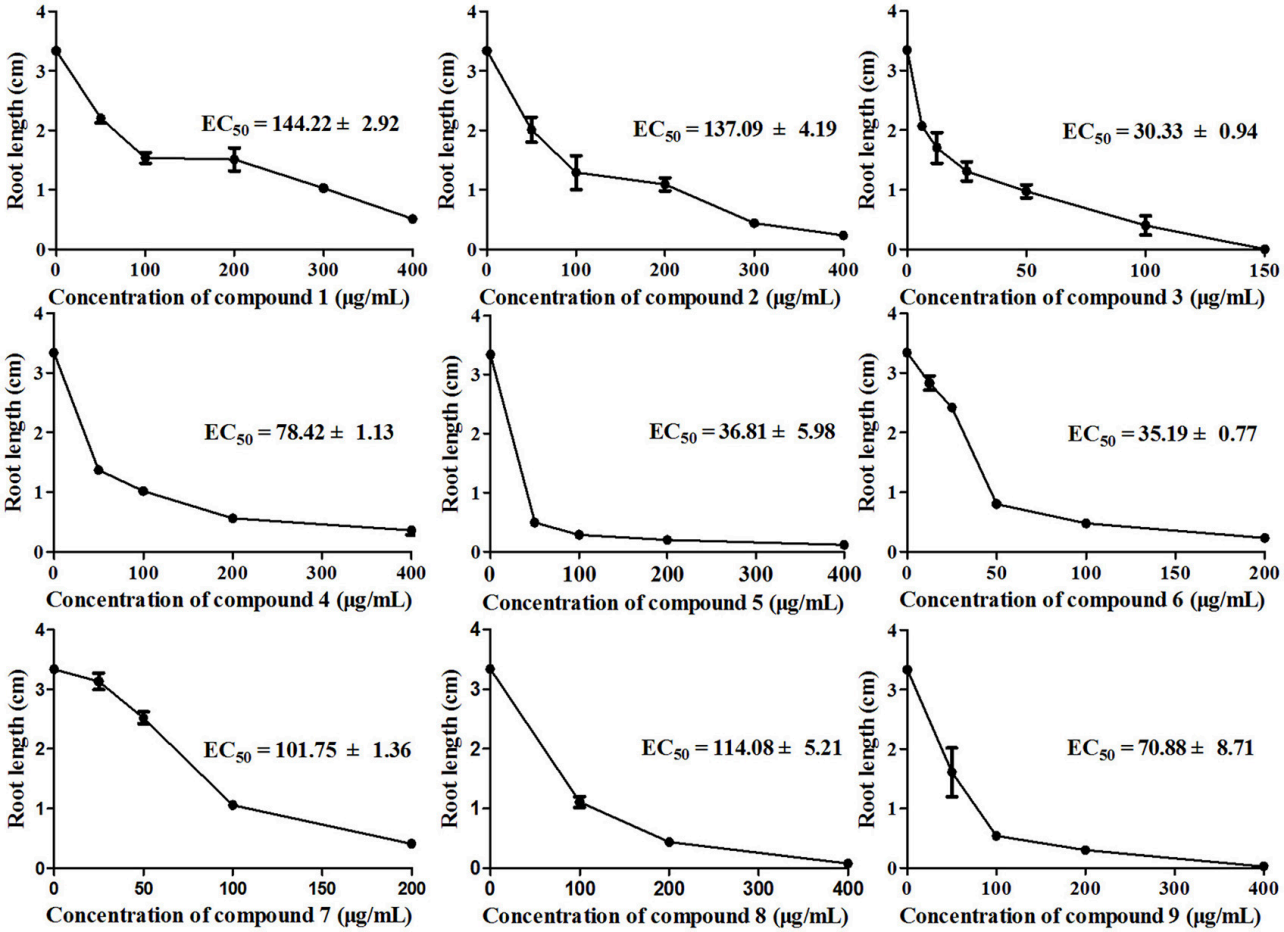
**Inhibition of *A. thaliana* root elongation by compounds 1–9 at different concentrations after 7 days of treatment**. There were three replicates for each plate; error bars represent standard error of the mean, *n* = 3.

To investigate how compounds **1–9** affect the roots of *A. thaliana*, we examined root elongation and root viability after treatment with these compounds at different concentrations. As shown in Figure 5, all tested samples exhibited different degrees of inhibitory activity of root elongation in a dose-dependent manner and, at the maximum concentration (400 μg/mL), all samples presented 100% inhibition. In this bioassay, compounds **3**, **5**, and **6** had significant inhibitory activities, with EC_50_ values of 30.33 ± 0.94, 36.81 ± 5.98, and 35.19 ± 0.77μg/mL, respectively. These results show a similar trend compared with those in previous studies in which sesquiterpenes inhibited root elongation more effectively than they inhibited seed germination (Anese et al., 2015).

**Figure 6:**
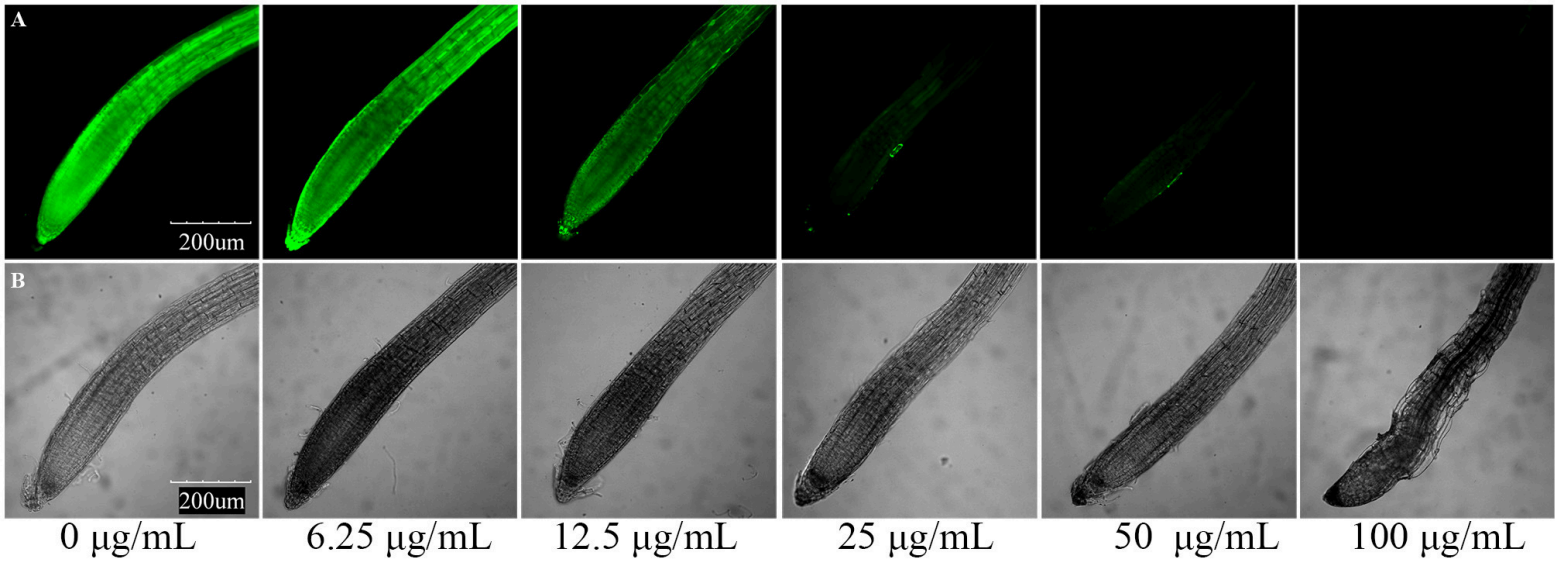
**Compound 3 induced root cell death in meristematic and CEZ cells of *A. thaliana*. (A)** Cell death proceeds with the sequential loss of FDA fluorescence. **(B)** Effect of compound **3** on the root tip cells of *A. thaliana*.

**Figure 7:**
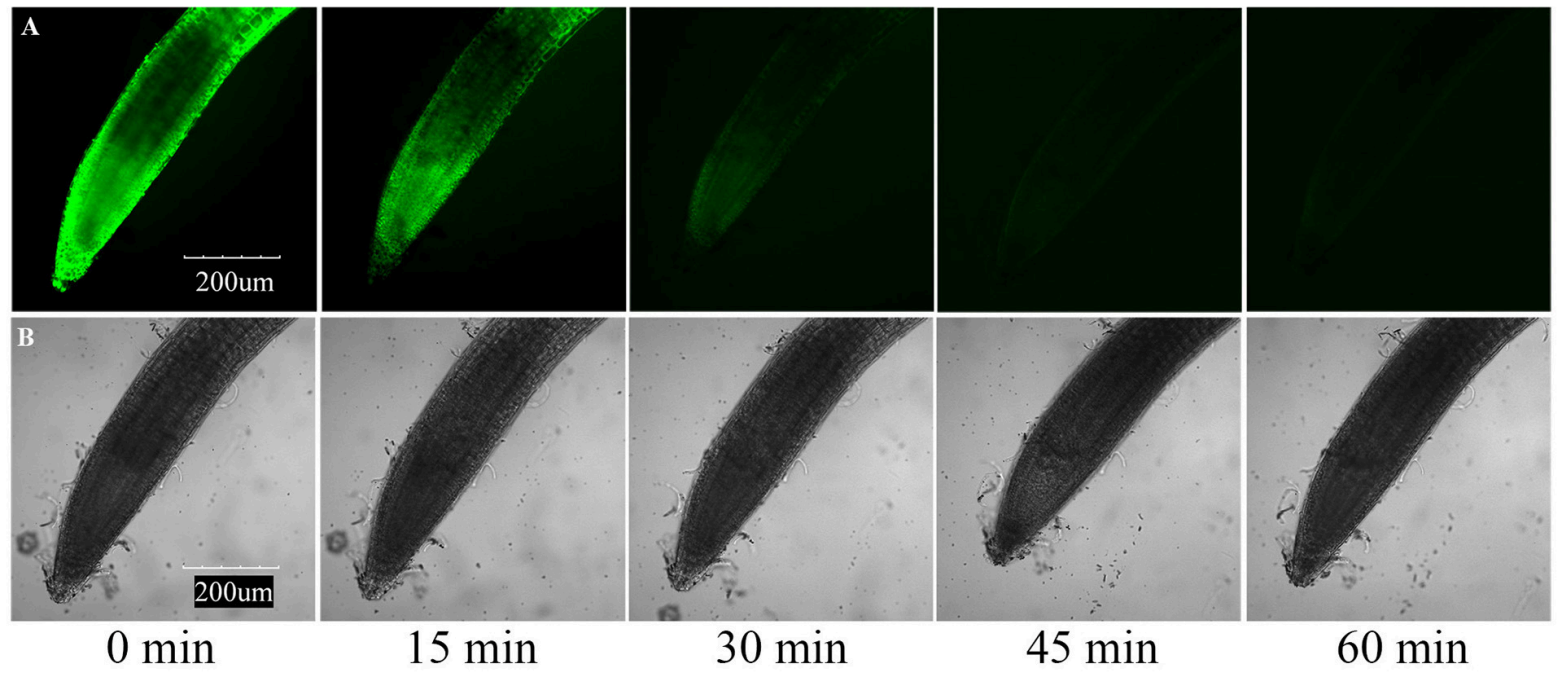
**Induction of cell death by 400 μg/mL compound 3 in meristematic and CEZ cells of *A. thaliana*. (A)** Cell death proceeds with sequential loss of FDA fluorescence. **(B)** Effect of compound **3** on the root tip cells of *A. thaliana*.

Furthermore, we determined the cell death of roots using the vital stain FDA, and found that the fluorescence of the root tip cells faded with increasing concentration of the applied compounds after 7 days of growth. The results of compounds induced root cell death are relatively similar to their inhibitory activities against root elongation. Compound **3** showed the strongest phytotoxic activity, although after 7 days of 25 μg/mL treatment, the fluorescence faded dramatically (Figure 6). To investigate the time-dependent phytotoxic activity of **3**, we further treated roots of *A. thaliana* seedlings grown for 5 days with 400 μg/mL of **3**, and found that, 15 min after treatment, the fluorescence of root tip cells began to fade, and the fluorescence faded dramatically 30 min after treatment (Figure 7). These results suggest that the inhibition of root elongation caused by these compounds was mostly due to the cell death at the root tips after treatment. However, the mechanism of these terpenoids inducing cell death in root tips was unknown.

The above results indicate that compounds **1**–**9** are phytotoxic chemicals in *L. cymbulifera*. Compound **3** was the most phytotoxic, exhibiting remarkable activity against both seedling growth and seed germination. The potential phytotoxic activities of these compounds mainly depended on their concentration and structure. Considering the structure-activity relationship in compounds **1–9** (Figures 2, 4, 5), the co-existence of a tri-substituted furan ring and OH-10 plays an essential role in the phytotoxic bioactivity (e.g., compound **3**), given that the absence of these two moieties caused a noticeable reduction in activity (e.g., compounds **1**, **2**, and **4**–**7**). Similarly, upon comparing the inhibitory activity between compounds **6** and **7**, it is possible that 8-OH weakens this activity. Furthermore, compound **3** showed stronger activity than compounds **6** and **8**, revealing that the furanoeremophilane-type sesquiterpenes are more active than the eremophilanolide-type ones, and simple eremophilane-type ones show the weakest activities. In addition, compounds **1** and **2** showed almost the same inhibitory activities against seed germination (EC_50_ of 144.22 ± 2.92 and 137.09 ± 4.19 μg/mL, respectively), indicating that their stereochemistry should not influence their activity. Together, these results suggest that the basic eremophilane structures, as well as the co-existence of the tri-substituted furan ring and the OH-10, appeared to be important for phytotoxicity.

### *L. cymbulifera* may release phytotoxic chemicals into rhizosphere soil to get competitive advantage

**Figure 8:**
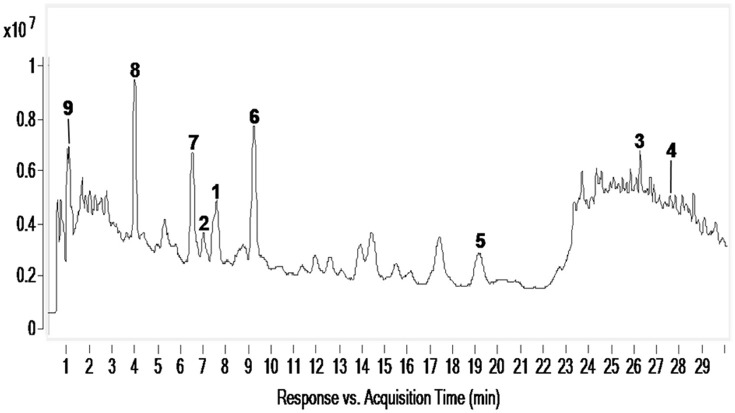
**UHPLC-MS analysis of MeOH extracts of *L. cymbulifera* rhizosphere soil**. Compounds **1**–**9** are shown in the total ion current (TIC) chromatogram with retention times of 7.607, 7.044, 26.388, 27.838, 19.259, 9.279, 6.576, 4.019, and 1.785 min, respectively.

To determine whether phytotoxic compounds **1**–**9** were released from the roots of *L. cymbulifera* into the surrounding rhizosphere soil, rhizosphere soil samples were collected, extracted with MeOH, and then analyzed by UHPLC-MS under the conditions described above. Compounds **1**–**9** in the rhizosphere soil samples were readily identified by comparing their retention times and MS/MS data with the isolated authentic sample standards (Table 2). Figure 8 shows the results of a UHPLC-MS chromatogram of the MeOH extracts of rhizosphere soil; the existence of potential phytotoxins **1**–**9** in the rhizosphere soil was confirmed. These findings indicate that *L. cymbulifera* has the potential to release phytotoxic chemicals **1–9** into the rhizosphere soil; these compounds might thus act synergistically to exert phytotoxic activity against the germination and root elongation of neighboring plants. It is likely that these potential phytotoxins identified in surrounding rhizosphere soil were released from the plant partly by root exudation or decomposition of plant root residue because numerous fibrous roots were found in rhizosphere soil during the process of collecting soil samples (Bertin et al., 2003). However, the actual process of release and the fate of these terpenoids under natural field conditions remains unclear.

**Table 2:** **Compounds 1–9 identified in the rhizosphere soil of *L. cymbulifera* by UHPLC-MS in positive ion mode**.

**Peak no**.	**t_R_(min)**	**Identified compound**	**Molecular formula**	**Molecular weight**	**Precursor Ion (m/z)**	**Collision energy (eV)**	**Characteristic fragment (m/z)**
1	1.785	**9**	C_10_H_18_O_2_	170	193	20	152, 135, 107
2	4.019	**8**	C_15_H_24_O_2_	236	237	20	219, 204, 189
3	6.576	**7**	C_15_H_22_O_4_	266	267	10	249, 231, 213
4	7.044	**2**	C_19_H_28_O_4_	320	321	10	303, 285, 275
5	7.607	**1**	C_19_H_28_O_4_	320	321	10	303, 285, 267
6	9.279	**6**	C_15_H_22_O_3_	250	251	20	233, 215, 187
7	19.259	**5**	C_15_H_16_O_3_	244	245	20	227, 209, 181
8	26.388	**3**	C_15_H_22_O_2_	234	235	10	217, 207, 189
9	27.838	**4**	C_15_H_17_O_3_	344	345	10	245, 227, 199

Compound **3** was the most potent phytotoxin in *L. cymbulifera*, and a quantitative analysis was therefore also carried out by UPLC-MS. A standard curve of **3** was obtained using linear regression. The results disclosed that the average concentration of **3** in rhizosphere soil was 3.44 μg/g. This suggested that *L. cymbulifera* might synthesize phytotoxic terpenoids continuously, by which they accumulate in surrounding rhizosphere soil and reach an effective concentration. Studies have shown that compound **3** might interfere with the enzymes involved in amino acid metabolism by reaction with pyridoxal at room temperature without any catalyst, due to the presence of its electron-rich tri-substituted furan ring (Iida et al., 2007; Torihata and Kuroda, 2008). Similarly, in our study, the phytotoxic activities of compound **3** mainly depend on the co-existence of the tri-substituted furan ring and the OH-10. These results provide some evidence that, one of the mechanisms of phytotoxic activity of compound **3** takes place via interference with the amino acid metabolism of other plants. However, there is a problem associated with **3** in terms of it acting as an active phytotoxin in the wild, namely, its instability. Its tri-substituted furan ring might be prone to reacting with electron-deficient reagents in the soil (Iida et al., 2007). Nevertheless, a recent study showed that, because of co-competitive sorption and preferential degradation, a mixture of phytotoxins exhibits greater persistence than single ones in the soil (Tharayil et al., 2008). Compound **3** might accumulate with other phytotoxins in rhizosphere soil, and the mixture of these phytotoxins would lead to greater bioavailability and a longer half-life. Additional studies are needed to obtain a better understanding of the mechanism of action of **3** and further research of **3** might lead it develop into a new eco-friendly natural herbicide.

### Compounds 1, 2, and 4-7 may derivate from compound 3

**Figure 9:**
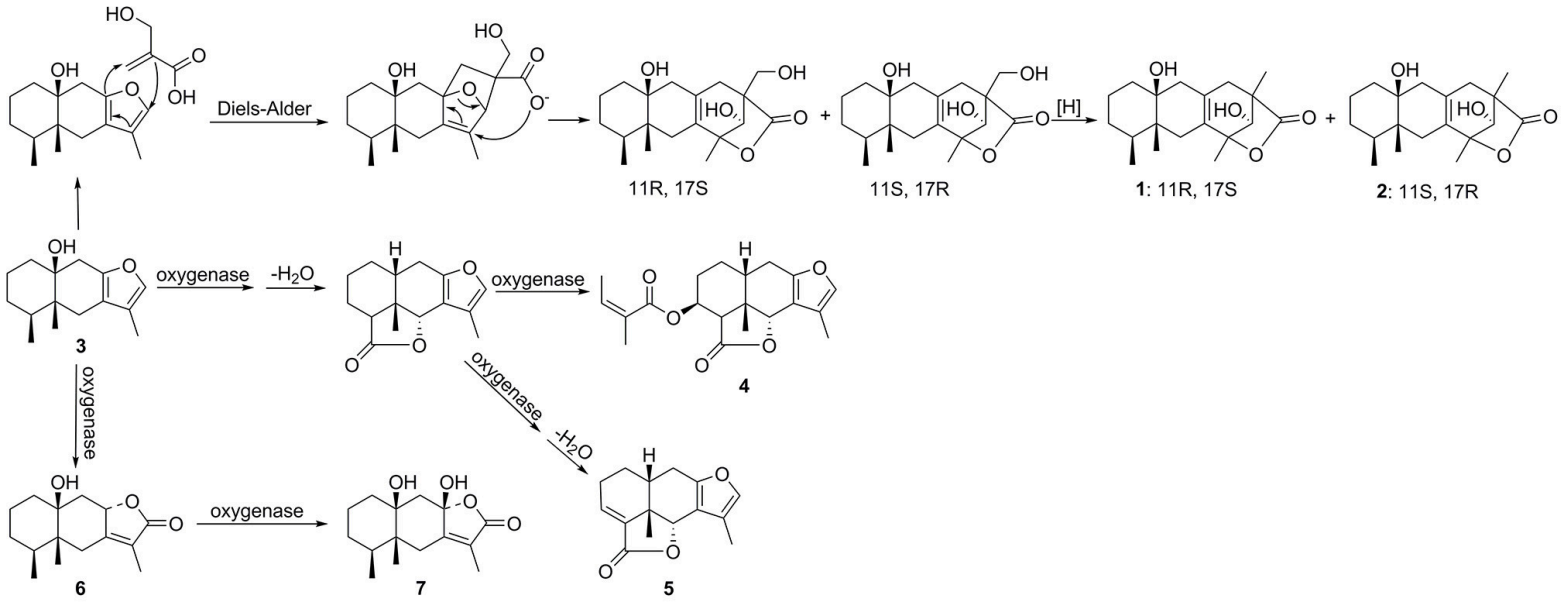
**Hypothesized biosynthetic pathway of compounds 1, 2, and 4–7**.

Notably, the compounds ligulacymirin A (**1**) and B (**2**) were isolated from *L. cymbulifera* roots for the first time and their structures were found to differ from those of other known eremophilane sesquiterpenes (Zhao et al., 1997; Chen et al., 2014). Figure 9 presents the hypothesis that the novel skeleton of **1** and **2** might be derived from compound **3**, a common eremophilane sesquiterpene in the same plant, followed by Diels-Alder reaction and subsequent oxidative modification. This finding makes a new addition to our understanding of eremophilane sesquiterpenes. It has been demonstrated that the Diels-Alder reaction can be catalyzed by natural Diels-Alderases from microorganisms, indicating that the endophytes in roots of *L. cymbulifera* might be involved in the biosynthesis of **1** and **2** (Hashimoto et al., 2015; Hashimoto and Kuzuyama, 2016). The production of **1** and **2** might be the result of co-evolution of *L. cymbulifera* and coexisting microorganisms in an unusual environment in which they faced unusual stresses. In this study, these two abundant compounds showed slight phytotoxic activities (Figures 4, 5), which may indicate that they have other main ecological roles.

Similarly, it is reasonable to deduce that the other eremophilane sesquiterpenes compounds **4**–**7** are also derived from compound **3** (Zhao et al., 2015). It is clear that, throughout the course of evolution, secondary metabolites in plants have gained numerous new ecological and physiological roles to secure optimal responses to challenges by biotic or abiotic stresses (Bertin et al., 2003; Walker et al., 2003). This has clearly been a useful strategy for plants in flexibly synthesizing secondary metabolites to endure a wider variety of stresses in a cost-effective biosynthetic manner (Neilson et al., 2013). Accordingly, we hypothesize that *L. cymbulifera* could continuously synthesize excessive **3** as the key intermediate in the biosynthetic pathway of eremophilane sesquiterpene derivatives for conversion into other phytotoxic active eremophilane sesquiterpenes (Figure 9). These phytotoxic chemicals would then display multiple ecological roles to face unusual stresses in the environment, including synergistic phytotoxic activity against neighboring plants (Siemens and Haugen, 2013).

## Conclusions

In conclusion, nine terpenoids (**1**–**9**) including two novel eremophilane sesquiterpene derivatives (**1** and **2)** were isolated from the roots of *L. cymbulifera*. Compounds **3**, **5**, and **6** exhibited significant phytotoxic activities, while **3** was the most phytotoxic chemical in *L. cymbulifera*. The average content of compound **3** in rhizosphere soil was 3.44 μg/g. These results indicate that terpenoids in *L. cymbulifera* roots might be released into the surrounding rhizosphere soil as phytotoxins. These phytotoxic terpenoids would synergistically interfere with the germination and root elongation of neighboring plants to help *L. cymbulifera* gain an advantage in the particular habitat. Additional studies will be performed to obtain a better understanding of the mechanisms of action associated with these phytotoxic terpenoids in the roots of *L. cymbulifera*.

## Author contributions

XH and GZ conceived the research and designed the experiments. JC and GZ performed the experiments, wrote the main manuscript and prepared figures and tables. XH, HA, and YZ revised the manuscript. All authors read and approved the version of the manuscript.

## Funding

This research was supported financially by Central Asian Drug Discovery, Development Center of Chinese Academy of Sciences (CAM201402 and CAM201302), Technological Leading Talent Project of Yunnan (2015HA020), and National Natural Science Foundation of China (31401313).

### Conflict of interest statement

The authors declare that the research was conducted in the absence of any commercial or financial relationships that could be construed as a potential conflict of interest.

## Supporting information

1D and 2D NMR, UV, IR, OR, and HR-ESIMS data of compounds **1** and **2** (Figures S1–S20).

## References

[B1] Aclinqu P. Benkoudier A. Massiot G. Le Men-Olivier L. (1991). Eremophilenolides from *Hertia cheirifolia*. Phytochemistry 30, 2083–2084. 10.1016/0031-9422(91)85074-A

[B2] Andolfi A. Zermane N. Cimmino A. Avolio F. Boari A. Vurro M.. (2013). Inuloxins A-D, phytotoxic bi-and tri-cyclic sesquiterpene lactones produced by Inula viscosa: potential for broomrapes and field dodder management. Phytochemistry 86, 112–120. 10.1016/j.phytochem.2012.10.003 23137725

[B3] Anese S. Jatoba L. J. Grisi P. U. Gualtieri S. C. J. Santos M. F. C. Berlinck R. G. S. (2015). Bioherbicidal activity of drimane sesquiterpenes from *Drimys brasiliensis* Miers roots. Ind. Crops Prod. 74, 28–35. 10.1016/j.indcrop.2015.04.042

[B4] Bertin C. Yang X. Weston L. A. (2003). The role of root exudates and allelochemicals in the rhizosphere. Plant Soil 256, 67–83. 10.1023/A:1026290508166

[B5] Cantrell C. L. Duke S. O. Fronczek F. R. Osbrink W. L. A. Mamonov L. K. Vassilyev J. I.. (2007). Phytotoxic Eremophilanes from *Ligularia macrophylla*. J. Agric. Food Chem. 55, 10656–10663. 10.1021/jf072548w 18044832

[B6] Chen J.-J. Chen C.-J. Yao X.-J. Jin X.-J. Gao K. (2014). Eremophilane-type sesquiterpenoids with diverse skeletons from *Ligularia sagitta*. J. Nat. Prod. 77, 1329–1335. 10.1021/np5003302 24913558

[B7] Cuenca M. D. R. Catalan C. A. N. Diaz J. G. Herz W. (1991). Monoterpenes and lignans from *Mikania saltensis*. J. Nat. Prod. 54, 1162–1164. 10.1021/np50076a047

[B8] Field B. Jordán F. Osbourn A. (2006). First encounters-deployment of defence-related natural products by plants. New Phytol. 172, 193–207. 10.1111/j.1469-8137.2006.01863.x 16995908

[B9] Hanai R. Gong X. Tori M. Kondo S. Otose K. Okamoto Y. (2005). Chemical and genetic study of *Ligularia tongolensis, Ligularia cymbulifera*, and *Ligularia atroviolacea* in the Hengduan mountains of China. Bull. Chem. Soc. Jpn. 78, 1302–1308. 10.1246/bcsj.78.1302

[B10] Hashimoto T. Hashimoto J. Teruya K. Hirano T. Shin-ya K. Ikeda H.. (2015). Biosynthesis of versipelostatin: identification of an enzyme-catalyzed [4+2]-cycloaddition required for macrocyclization of spirotetronate-containing polyketides. J. Am. Chem. Soc. 137, 572–575. 10.1021/ja510711x 25551461 PMC4308742

[B11] Hashimoto T. Kuzuyama T. (2016). Mechanistic insights into Diels-Alder reactions in natural product biosynthesis. Curr. Opin. Chem. Biol. 35, 117–123. 10.1016/j.cbpa.2016.09.015 27697700

[B12] Huang H. Morgan C. M. Asolkar R. N. Koivunen M. E. Marrone P. G. (2010). Phytotoxicity of sarmentine isolated from long pepper (*Piper longum*) Fruit. J. Agric. Food Chem. 58, 9994–10000. 10.1021/jf102087c 20839888

[B13] Iida K. Mitani M. Kuroda C. (2007). Fixation of natural furanoeremophilane by Diels-Alder reaction. Bull. Chem. Soc. Jpn. 80, 966–971. 10.1246/bcsj.80.966

[B14] Jennings P. W. Hurley J. C. Reeder S. K. Holian A. Lee P. Caughlan C. N.. (1976). Isolation and structure determination of the second toxic constituent from *Tetradymia glabrata*. J. Org. Chem. 41, 4078–4081. 10.1021/jo00888a006 1003262

[B15] Kojima K. Hatano K. Ondognij P. Munaagin B. Oihara Y. (1997). Absolute structure of eremophilenolide from *Solidago dahurica*. Chem. Pharm. Bull. 45, 1875–1876. 10.1248/cpb.45.1875

[B16] Kuroda C. Hanai R. Nagano H. Tori M. Gong X. (2012). Diversity of furanoeremophilanes in major *Ligularia* species in the Hengduan mountains. Nat. Prod. Commun. 7, 539–548. 22574462

[B17] Kuroda C. Murae T. Tada M. Nagano H. Tsuyuki T. Takahashi T. (1982). 3β,6β-Bis(acyloxy) furanoeremophilan-14-als and -14-oic acids from *Syneilesis palmata* (Thunb.) Maxim. Bull. Chem. Soc. Jpn. 55, 1228–1233. 10.1246/bcsj.55.1228

[B18] Li Y. S. Wang Z. T. Zhang M. Zhou H. Chen J. J. Luo S. D. (2004). New eremophilane-type sesquiterpenes from *Ligularia lapathifolia*. Planta Med. 70, 239–243. 10.1055/s-2004-815541 15114501

[B19] Liu C.-M. Fei D.-Q. Wu Q.-H. Gao K. (2006). Bisabolane sesquiterpenes from the roots of *Ligularia cymbulifera*. J. Nat. Prod. 69, 695–699. 10.1021/np0505061 16643057

[B20] Liu C.-M. Wang H.-X. Wei S.-L. Gao K. (2008). Pyrrolizidine alkaloids and bisabolane sesquiterpenes from the roots of *Ligularia cymbulifera*. Helv. Chim. Acta 91, 308–316. 10.1002/hlca.200890036

[B21] Macías F. A. Oliveros-Bastidas A. Marín D. Carrera C. Chinchilla N. Molinillo J. M. G. (2008). Plant biocommunicators: their phytotoxicity, degradation studies and potential use as herbicide models. Phytochem. Rev. 7, 179–194. 10.1007/s11101-007-9062-4

[B22] Macías F. A. Oliveros-Bastidas A. Marín D. Chinchilla N. Castellano D. Molinillo J. M. G. (2014). Evidence for an allelopathic interaction between rye and wild oats. J. Agric. Food Chem. 62, 9450–9457. 10.1021/jf503840d 25233257

[B23] Masi M. Meyer S. Cimmino A. Clement S. Black B. Evidente A. (2014). Pyrenophoric acids B and C, two new phytotoxic sesquiterpenoids produced by *Pyrenophora semeniperda*. J. Agric. Food Chem. 62, 10304–10311. 10.1021/jf5035515 25264583

[B24] Miranda M. A. F. M. Varela R. M. Torres A. Molinillo J. M. G. Gualtieri S. C. J. Macías F. A. (2015). Phytotoxins from *Tithonia diversifolia*. J. Nat. Prod. 78, 1083–1092. 10.1021/acs.jnatprod.5b00040 25879678

[B25] Neilson E. H. Goodger J. Q. D. Woodrow I. E. Møeller B. L. (2013). Plant chemical defense: at what cost? Trends Plant Sci. 18, 250–258. 10.1016/j.tplants.2013.01.001 23415056

[B26] Saito Y. (2012). Chemical and genetic diversity of *Ligularia* plants collected in the Hengduan Mountains, China. Yakugaku Zasshi 132, 1451–1459. 10.1248/yakushi.12-00207 23208053

[B27] Savona G. Piozzi F. De la Torre M. C. Servettaz O. Rodriguez B. (1987). A valencane sesquiterpenoid from *Teucrium carolipaui*. Phytochemistry 26, 571–572. 10.1016/S0031-9422(00)81458-2

[B28] Seigler D. S. (1996). Chemistry and mechanisms of allelopathic interactions. Agron. J. 88, 876–885. 10.2134/agronj1996.00021962003600060006x

[B29] Siemens D. H. Haugen R. (2013). Plant chemical defense allocation constrains evolution of tolerance to community change across a range boundary. Ecol. Evol. 3, 4339–4347. 10.1002/ece3.657 24340176 PMC3856735

[B30] Tharayil N. Bhowmik P. C. Xing B. (2008). Bioavailability of allelochemicals as affected by companion compounds in soil matrices. J. Agric. Food Chem. 56, 3706–3713. 10.1021/jf073310a 18435537

[B31] Tori M. (2016). Diversity of plants belonging to the genus *Ligularia* (Asteraceae) based on terpenoids and synthetic studies on some terpenoids. Yakugaku Zasshi 136, 309–327. 10.1248/yakushi.15-00238 26831809

[B32] Torihata A. Kuroda C. (2008). Reaction of Furanoeremophilans with Pyridoxal. Nat. Prod. Commun. 3, 1659–1662. 14214072

[B33] Walker T. S. Bais H. P. Grotewold E. Vivanco J. M. (2003). Root exudation and rhizosphere biology. Plant Physiol. 132, 44–51. 10.1104/pp.102.019661 12746510 PMC1540314

[B34] Wang Y. Luo S. H. Hua J. Liu Y. Jing S. X. Li X. N.. (2015). Capitate glandular trichomes of *Paragutzlaffia henryi* harbor new phytotoxic labdane diterpenoids. J. Agric. Food Chem. 63, 10004–10012. 10.1021/acs.jafc.5b04113 26513276

[B35] Wu Y.-X. Chen Y.-J. Liu C.-M. Gao K. (2012). Four new sesquiterpenoids from *Ligularia cymbulifera*. J. Asian Nat. Prod. Res. 14, 1130–1136. 10.1080/10286020.2012.733002 23088569

[B36] Yang J.-L. Wang R. Shi Y.-P. (2011). Phytochemicals and biological activities of *Ligularia* species. Nat. Prod. Bioprospect. 1, 1–24. 10.1007/s13659-011-0003-y

[B37] Zhao G. Cao Z. Zhang W. Zhao H. (2015). The sesquiterpenoids and their chemotaxonomic implications in *Senecio L*. (Asteraceae). Biochem. Syst. Ecol. 59, 340–347. 10.1016/j.bse.2015.02.001

[B38] Zhao Y. Parsons S. Smart B. A. Tan R. Jia Z. Sun H. (1997). Eremophilane derivatives with a novel carbon skeleton from *Ligularia veitchiana*. Tetrahedron 53, 6195–6208. 10.1016/S0040-4020(97)00276-7

[B39] Zheng G. Jia Y. Zhao X. Zhang F. Luo S. Li S. (2012). o-Coumaric acid from invasive *Eupatorium adenophorum* is a potent phytotoxin. Chemoecology 22, 131–138. 10.1007/s00049-012-0105-y

