# Supplementary material for: Phytotoxic Terpenoids from Ligularia cymbulifera Roots
Source: Front Plant Sci. 2017 Jan 9;7:2033. doi: 10.3389/fpls.2016.02033 (PMC5221121; doi:10.3389/fpls.2016.02033)
Supplement: Supplementary file 1 [file Image1.PDF]

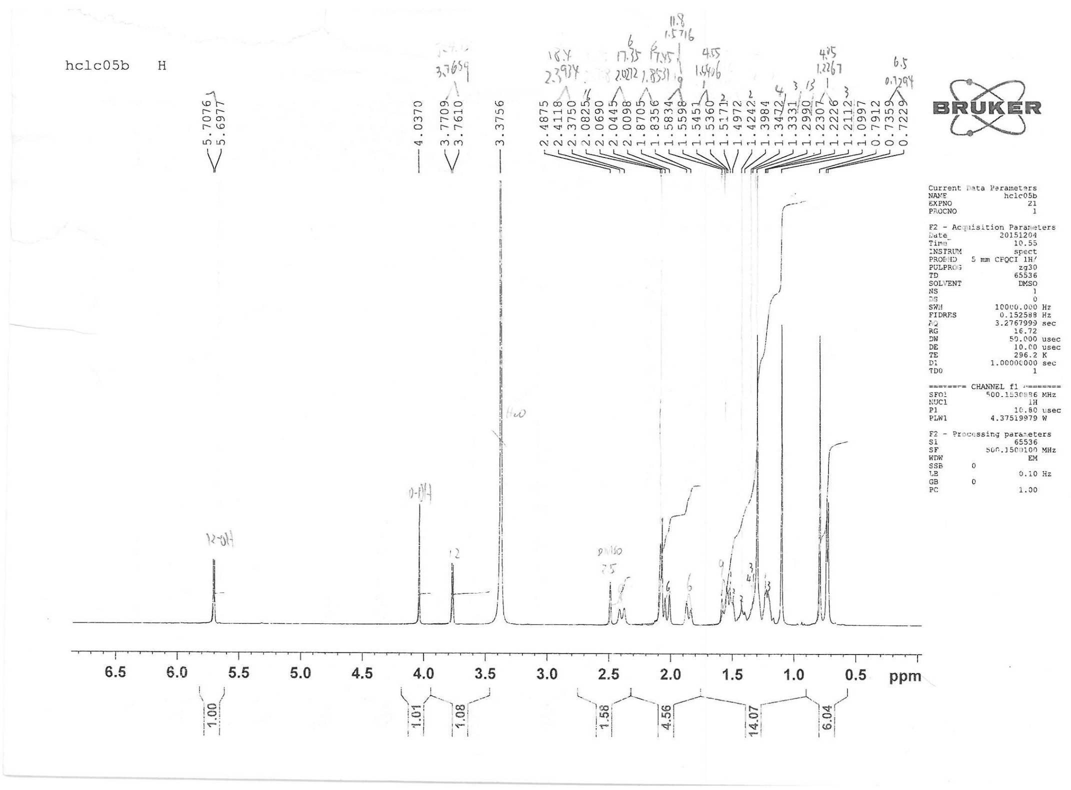

Figure S1. ^{1}H-NMR of **1.**

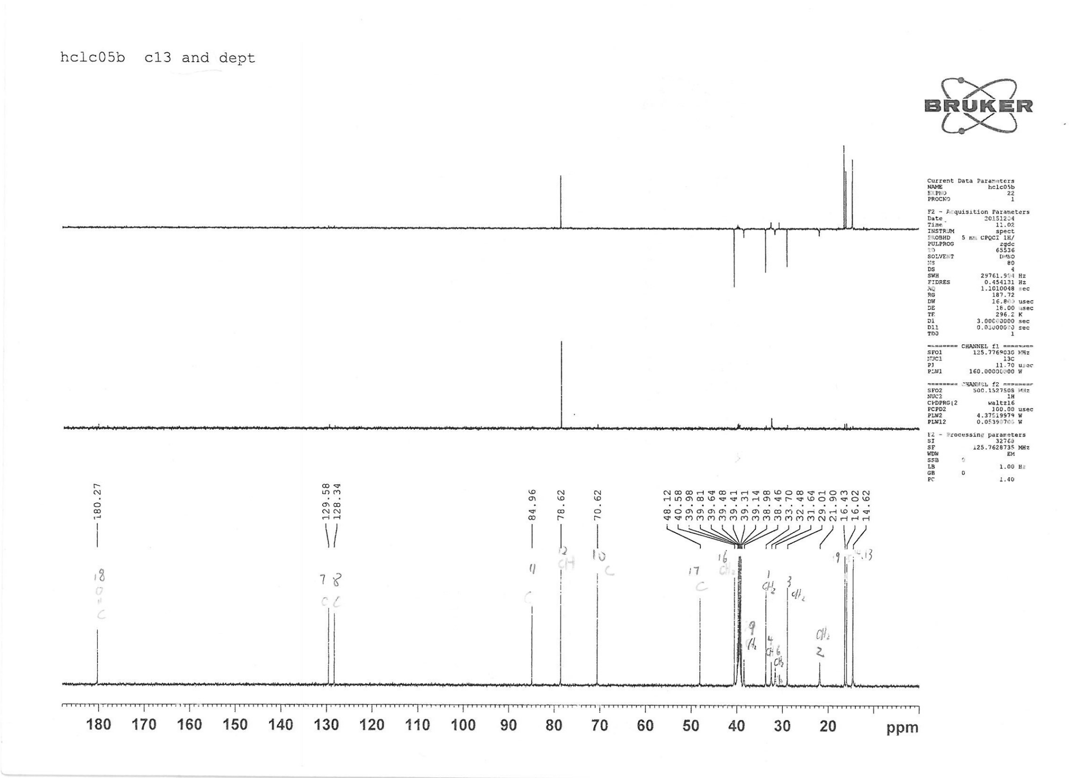

Figure S2. ^{13}C-NMR and DEPT of **1.**

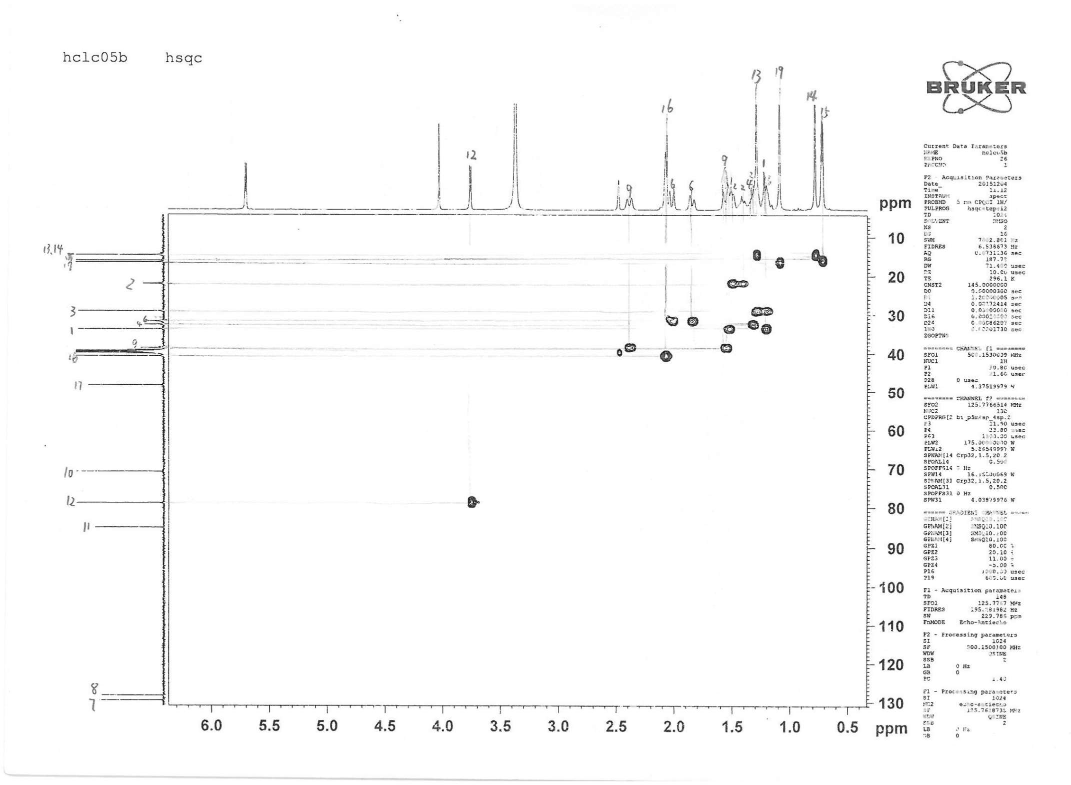

Figure S3. HSQC of **1.**

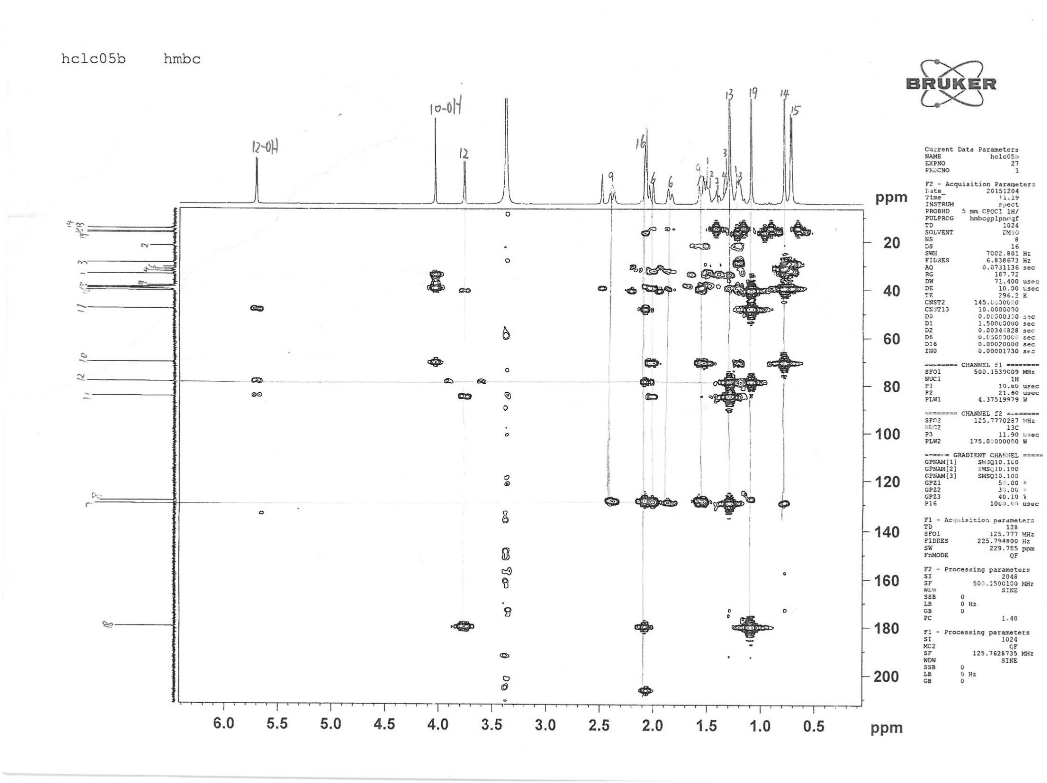

Figure S4. HMBC of **1.**

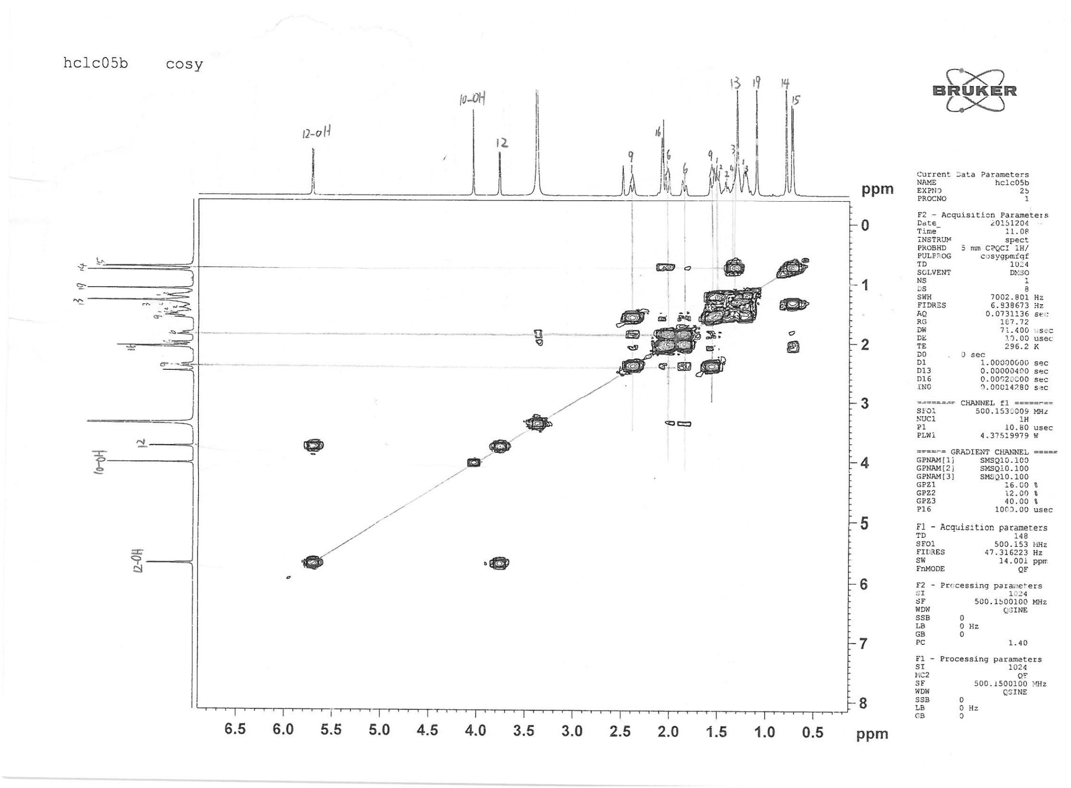

Figure S5. ^{1}H-^{1}H COSY of **1.**

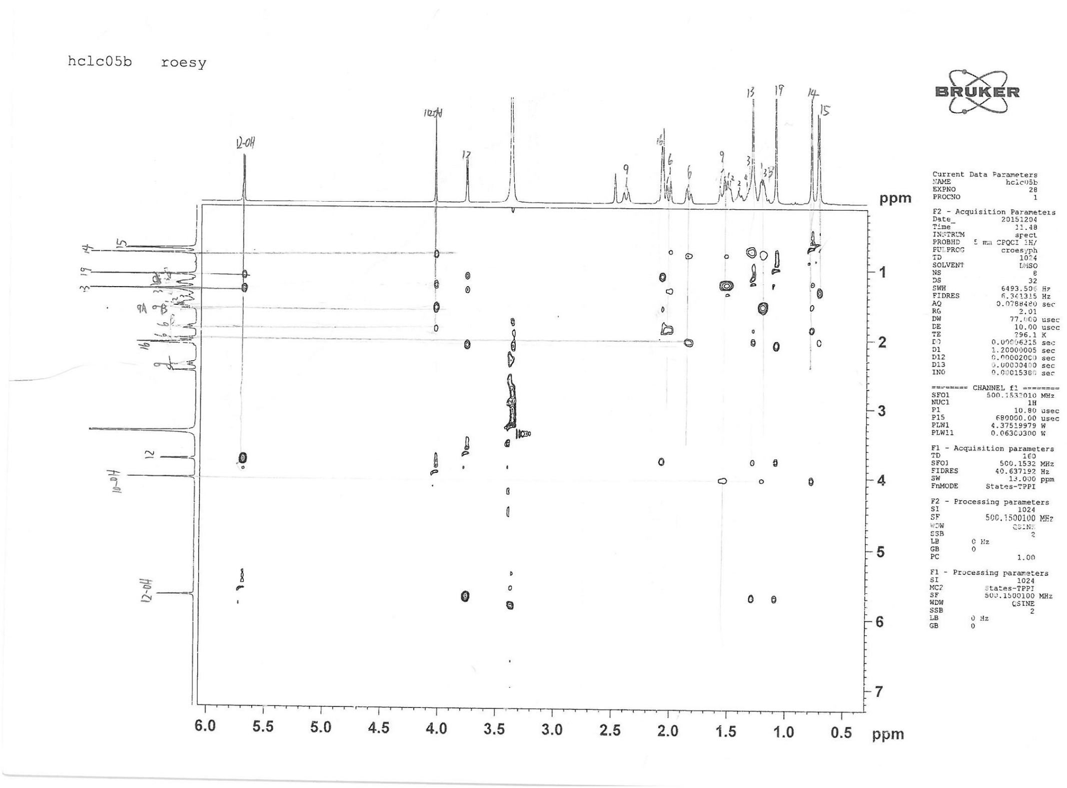

Figure S6. ROESY of **1.**

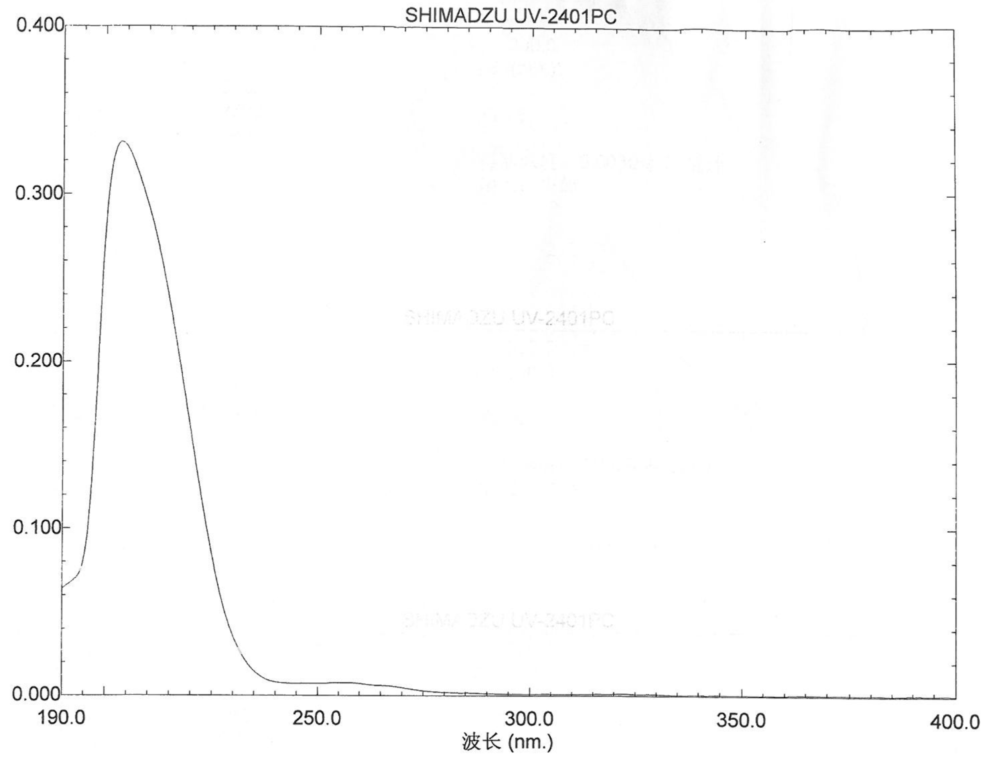

文件名:　HCLC-05

HCLC-05

样品浓度：0.0255毫克/毫升
溶剂：甲醇

创建于:　20:10　16-01-25
数据:　原始

测量模式:　Abs.
扫描速度:　中速
狭缝:　5.0
采样间隔:　0.2

否.	波长 (nm.)	Abs.
1	203.60	0.3310

Figure S7. UV of **1.**

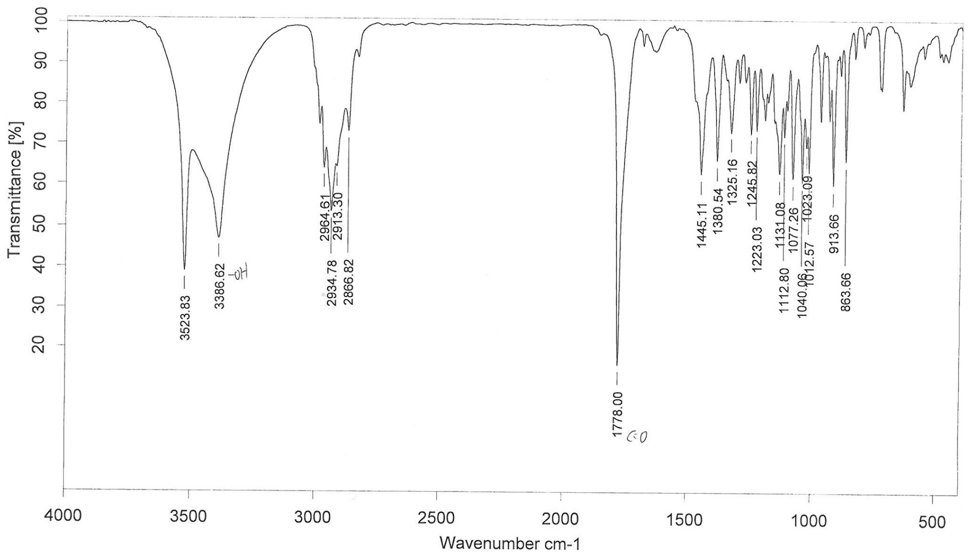

Sample : hclc-05	Frequency Range : 399.246 - 3996.32	Measured on : 14/01/2016	
Technique : KBr压片	Resolution : 4	Instrument : Tensor27	Sample Scans : 16
Customer : 160114IR4	Zerofilling : 2	Acquisition : Double Sided,For	

Figure S8. IR of **1.**

Optical rotation measurement

Model : P-1020 (A060460638)

No.	Sample	Mode	Data	Monitor<br>Blank	Temp.<br>Cell<br>Temp Point	Date<br>Comment<br>Sample Name	Light<br>Filter<br>Operator	Cycle Time<br>Integ Time
No.1	10 (1/3)	Sp.Rot	21.1760	0.0036<br>0.0000	18.5<br>10.00<br>Cell	Sun Jan 17 15:09:02 2016<br>0.00170g/mL MeOH<br>HCLC-05	Na<br>589nm	2 sec<br>10 sec
No.2	10 (2/3)	Sp.Rot	20.0000	0.0034<br>0.0000	18.5<br>10.00<br>Cell	Sun Jan 17 15:09:15 2016<br>0.00170g/mL MeOH<br>HCLC-05	Na<br>589nm	2 sec<br>10 sec +20.0000°
No.3	10 (3/3)	Sp.Rot	18.8240	0.0032<br>0.0000	18.5<br>10.00<br>Cell	Sun Jan 17 15:09:28 2016<br>0.00170g/mL MeOH<br>HCLC-05	Na<br>589nm	2 sec<br>10 sec

2263 [illegible]

Figure S9. OR of **1.**

Qualitative Analysis Report

**Data Filename**	hclc-05.d	**Sample Name**	hclc-05
**Sample Type**	Sample	**Position**	P1-B1
**Instrument Name**	Instrument 1	**User Name**	
**Acq Method**	SIBU.m	**Acquired Time**	2/26/2016 10:58:00 AM
**IRM Calibration Status**	Success	**DA Method**	ESI+.m
**Comment**			

Sample Group **Info.**
Acquisition SW 6200 series TOF/6500 series
Version Q-TOF B.05.01 (B5125.2)

User Spectra

Fragmentor Voltage	Collision Energy	Ionization Mode
135	0	ESI

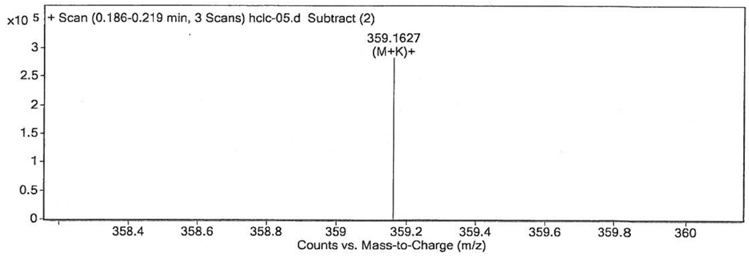

Peak List

m/z	z	Abund	Formula	Ion
303.1961	1	125594.73		
343.1887	1	192905.38		
359.1627	1	283694.78	C19 H28 O4	(M+K)+
360.1659	1	56516.08	C19 H28 O4	(M+K)+
366.2644	1	74570.24		
663.3891	1	296258.13		
664.3919	1	115709.7		
679.3619	1	64949.3		

Formula Calculator Element Limits

Element	Min	Max
C	3	60
H	0	120
O	0	30

Formula Calculator Results

Formula	CalculatedMass	CalculatedMz	Mz	Diff. (mDa)	Diff. (ppm)	DBE
C19 H28 O4	320.1988	359.1619	359.1627	-0.8	-2.4	6.0000

--- End Of Report ---

Figure S10. HR-ESIMS of **1.**

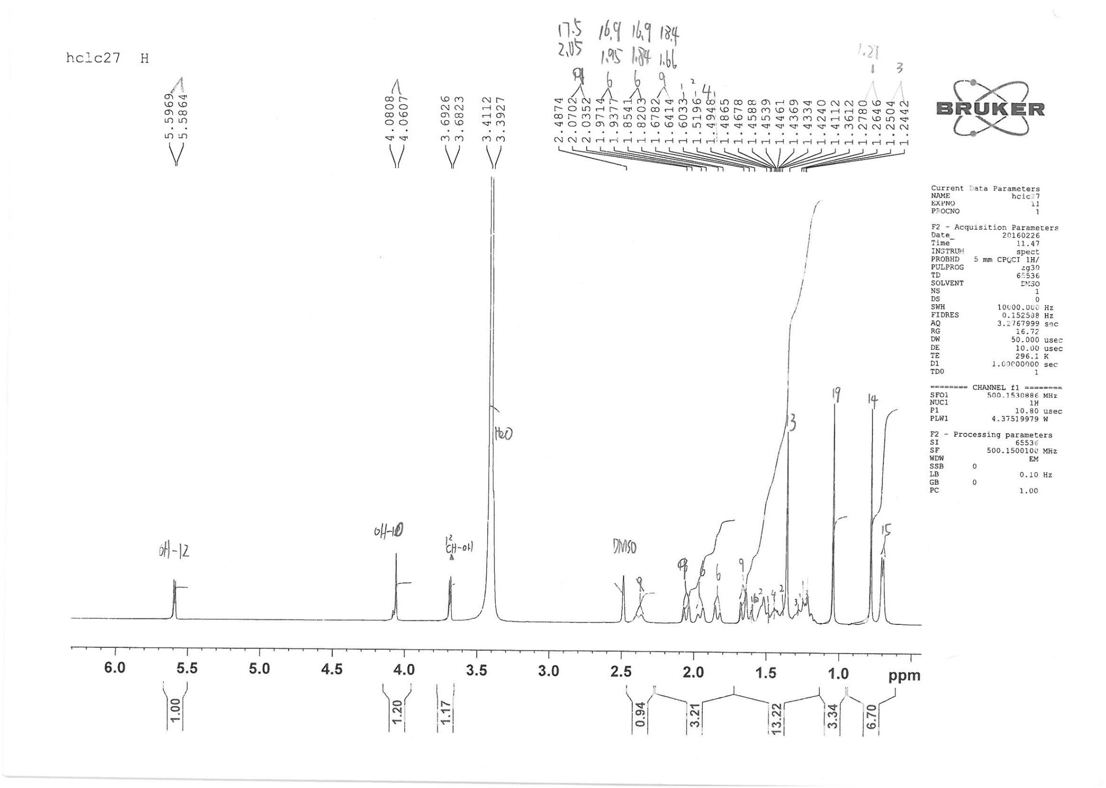

Figure S11. ^{1}H-NMR of **2.**

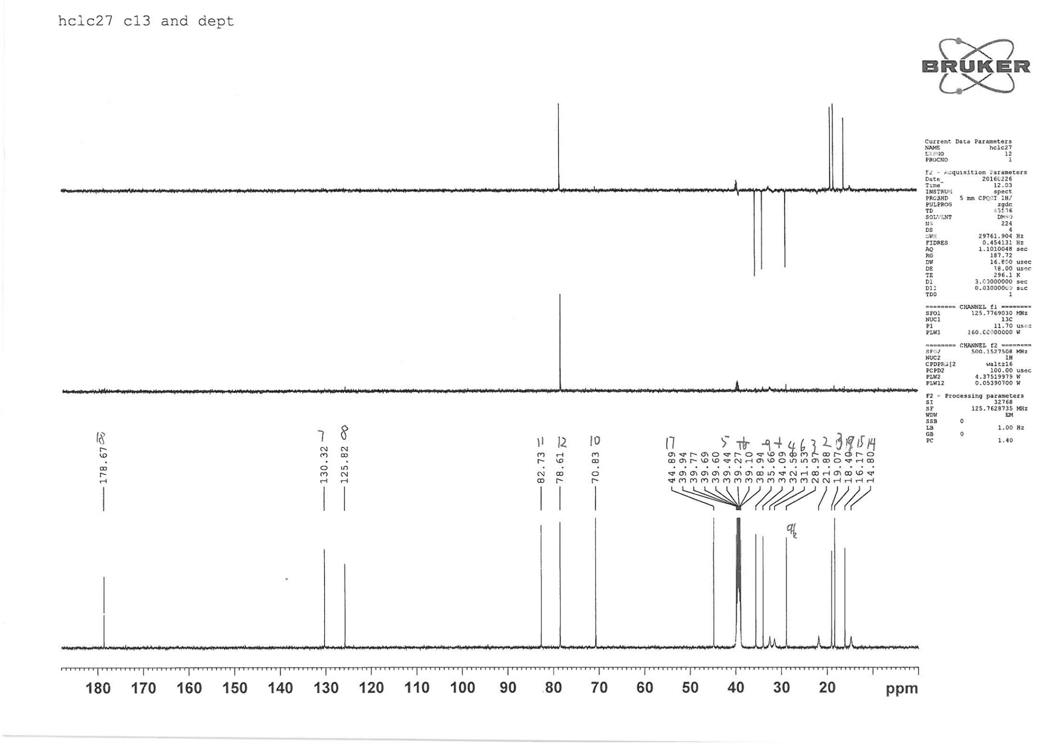

Figure S12. ^{13}C-NMR and DEPT of **2.**

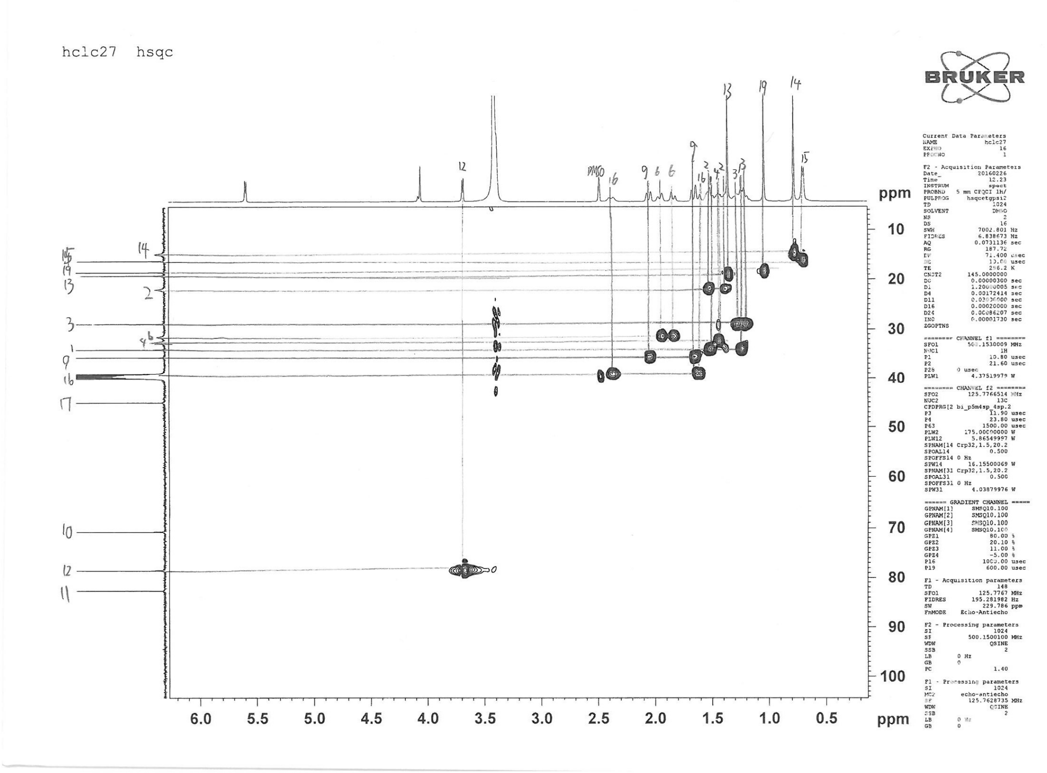

Figure S13. HSQC of **2.**

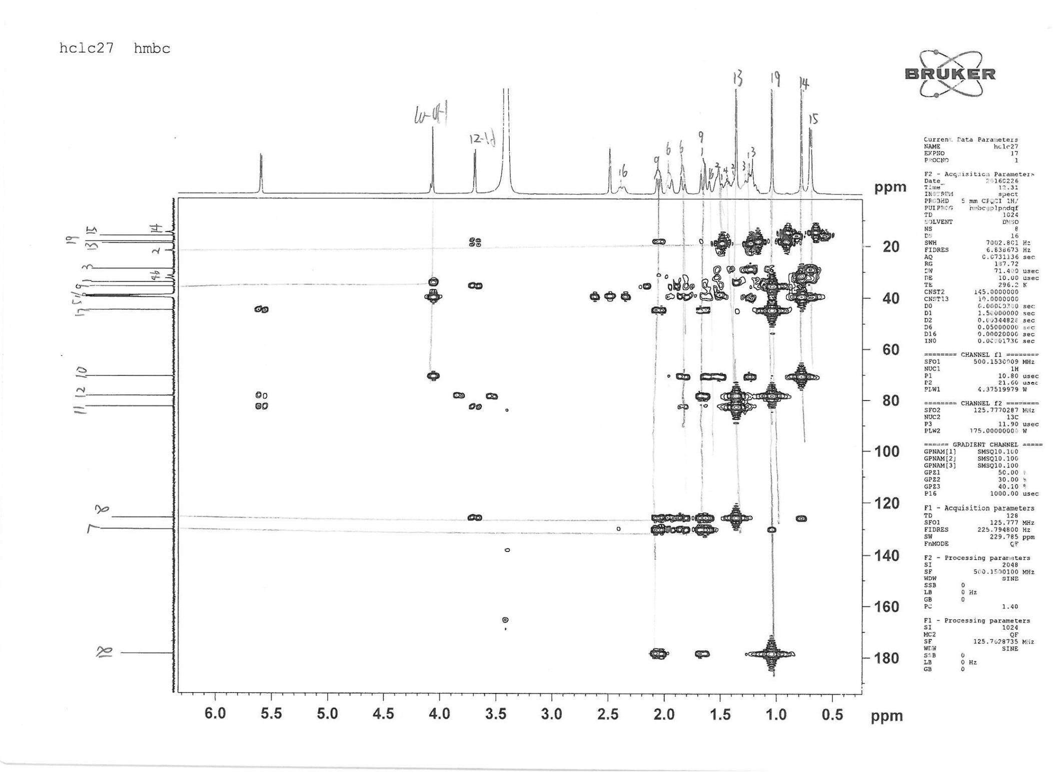

Figure S14. HMBC of **2.**

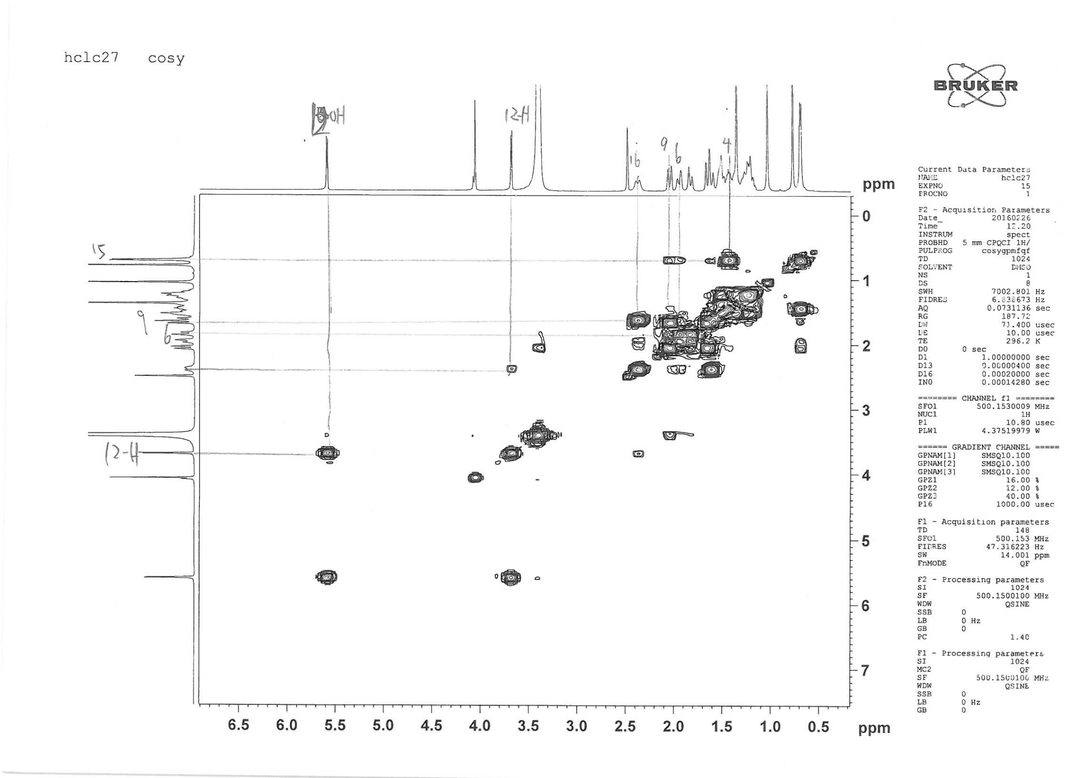

Figure S15. ^{1}H-^{1}H COSY of **2.**

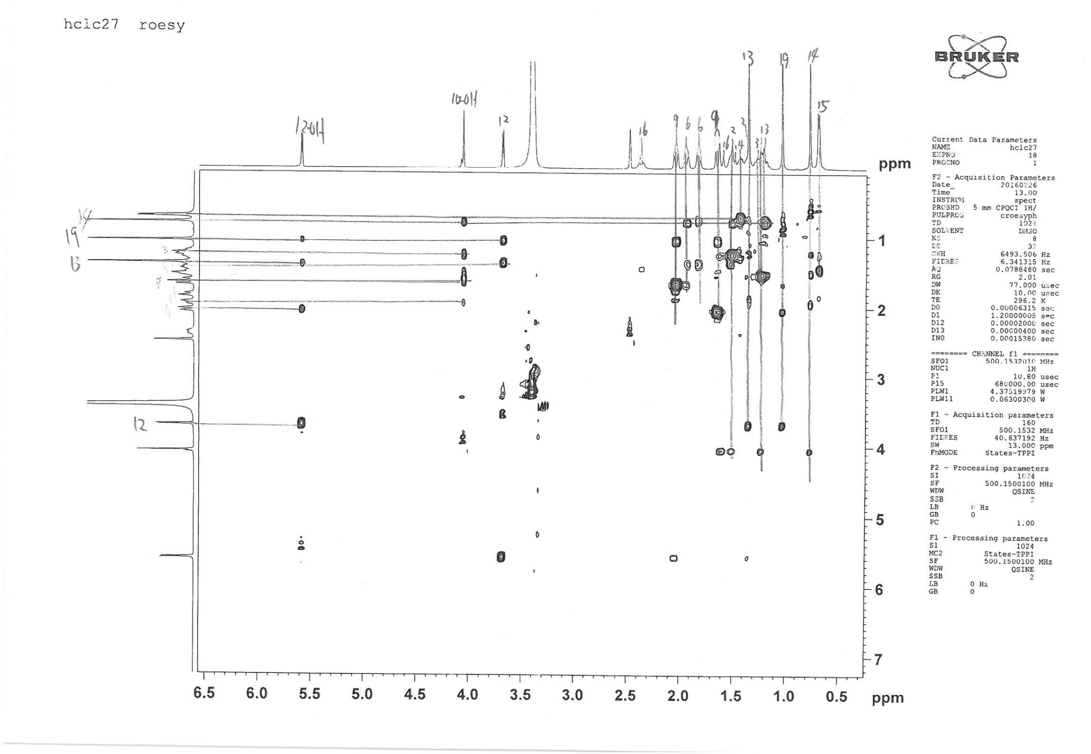

Figure S16. ROESY of **2.**

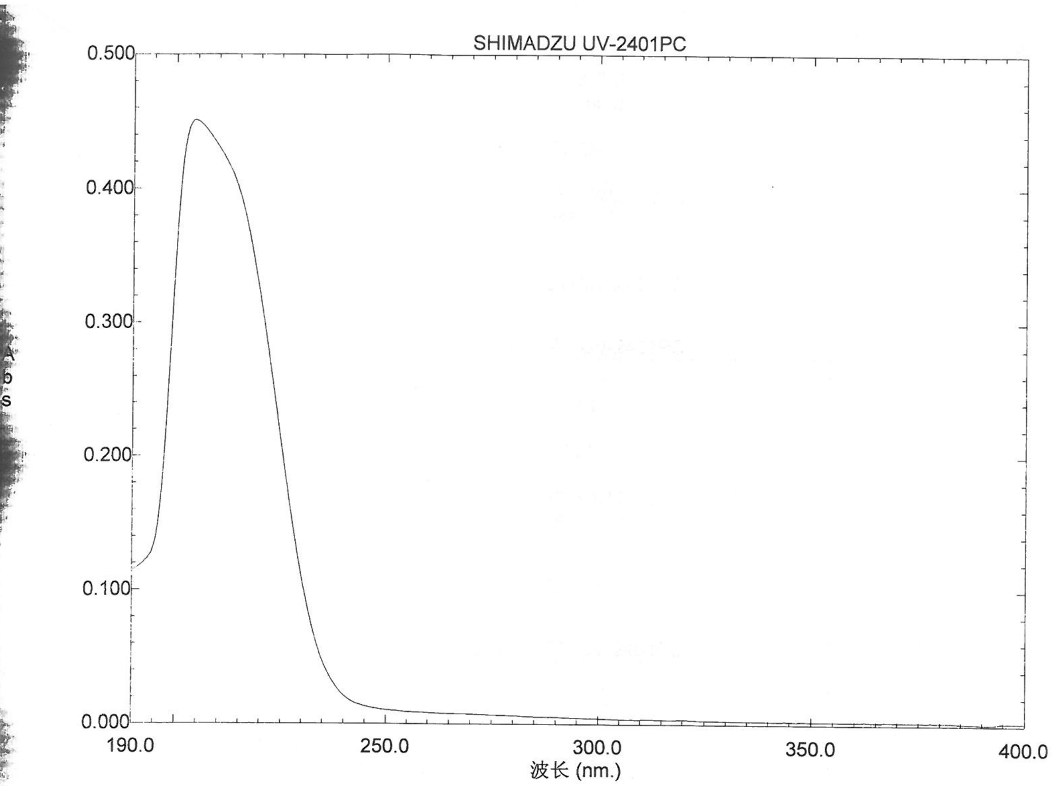

文件名: HCLC-27

HCLC-27———

样品浓度：0.0350毫克/毫升
溶剂：甲醇

创建于: 14:53 16-03-02
数据: 原始

测量模式: Abs.
扫描速度: 中速
狭缝: 5.0
采样间隔: 0.5

否.	波长 (nm.)	Abs.
1	426.50	0.0031
2	204.50	0.4510

[illegible]

Figure S17. UV of **2.**

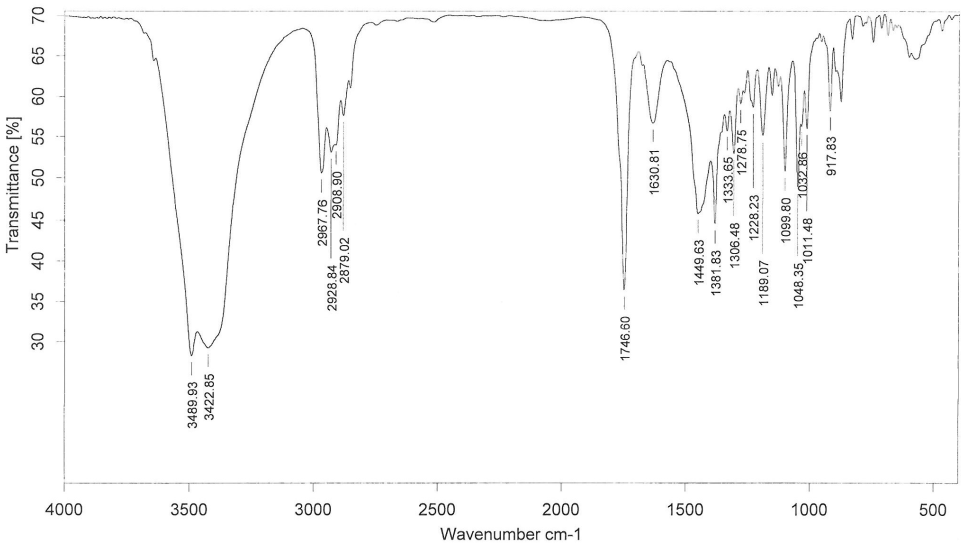

Sample : hclc-27	Frequency Range : 399.246 - 3996.32	Measured on : 25/02/2016	
Technique : KBr压片	Resolution : 4	Instrument : Tensor27	Sample Scans : 16
Customer : 160226IR0	Zerofilling : 2	Acquisition : Double Sided,For	

Figure S18. IR of **2.**

Optical rotation measurement

Model : P-1020 (A060460638)

No.	Sample	Mode	Data	Monitor<br>Blank	Temp.<br>Cell<br>Temp Point	Date<br>Comment<br>Sample Name	Light<br>Filter<br>Operator	Cycle Time<br>Integ Time
No.1	4 (1/3)	Sp.Rot	23.0000	0.0023<br>0.0000	19.5<br>10.00<br>Cell	Mon Feb 29 17:34:43 2016<br>0.00100g/mL MeOH<br>HCLC-27	Na<br>589nm	2 sec<br>10 sec
No.2	4 (2/3)	Sp.Rot	26.0000	0.0026<br>0.0000	19.4<br>10.00<br>Cell	Mon Feb 29 17:34:57 2016<br>0.00100g/mL MeOH<br>HCLC-27	Na<br>589nm	2 sec<br>10 sec
No.3	4 (3/3)	Sp.Rot	24.0000	0.0024<br>0.0000	19.4<br>10.00<br>Cell	Mon Feb 29 17:35:10 2016<br>0.00100g/mL MeOH<br>HCLC-27	Na<br>589nm	2 sec<br>10 sec

+24.3333°

Figure S19. OR of **2.**

Qualitative Analysis Report

**Data Filename**	hclc-27.d	**Sample Name**	hclc-27
**Sample Type**	Sample	**Position**	P1-B2
**Instrument Name**	Instrument 1	**User Name**	
**Acq Method**	SIBU.m	**Acquired Time**	2/26/2016 10:59:21 AM
**IRM Calibration Status**	Success	**DA Method**	ESI+.m
**Comment**			

Sample Group **Info.**

Acquisition SW Version 6200 series TOF/6500 series Q-TOF B.05.01 (B5125.2)

User Spectra

Fragmentor Voltage	Collision Energy	Ionization Mode
135	0	ESI

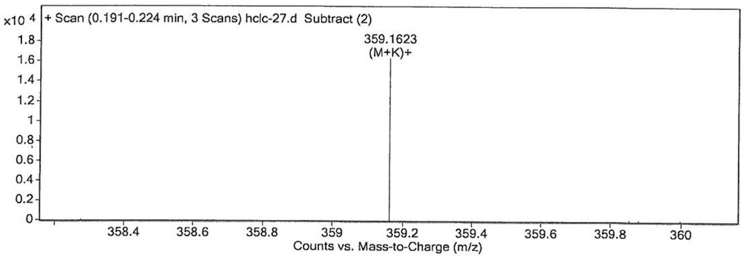

Peak List

m/z	z	Abund	Formula	Ion
164.1073	1	3324.31		
309.161	1	6159.41		
338.2329	1	6887.17		
341.1856	1	4293.88		
359.1623	1	16361.1	C19 H28 O4	(M+K)+
363.1675	1	3153.9		
379.1423	1	5723.67		
409.1535	1	4734.46		

Formula Calculator Element Limits

Element	Min	Max
C	3	60
H	0	120
O	0	30

Formula Calculator Results

Formula	CalculatedMass	CalculatedMz	Mz	Diff. (mDa)	Diff. (ppm)	DBE
C19 H28 O4	320.1988	359.1619	359.1623	-0.3	-1.0	6.0000

--- End Of Report ---

Figure S20. HR-ESIMS of **2.**
